# Theoretical Framework, Technical Evolution, and Future Prospects of Cross-Modal Mapping and Controllable Image Generation Under Multi-Source Heterogeneous Collaboration

**DOI:** 10.3390/s26102972

**Published:** 2026-05-08

**Authors:** Mingju Chen, Zhihao Lin, Xiaofei Song, Yangming Luo, Xueyang Duan, Senyuan Li, Chen Xie

**Affiliations:** 1 School of Automation and Information Engineering, Sichuan University of Science and Engineering, Yibin 644005, China; chenmingju@suse.edu.cn (M.C.); 323085404418@stu.suse.edu.cn (X.S.); 324085404516@stu.suse.edu.cn (Y.L.); 324085404203@stu.suse.edu.cn (X.D.); 323085404108@stu.suse.edu.cn (S.L.); 324085404528@stu.suse.edu.cn (C.X.); 2 Intelligent Perception and Control Key Laboratory of Sichuan Province, Sichuan University of Science and Engineering, Yibin 644005, China

**Keywords:** diffusion models, controllable image generation, cross-modal mapping, multi-source heterogeneous collaboration, technical evolution, architectural decoupling, performance game analysis

## Abstract

The rapid evolution of diffusion models has shifted visual synthesis from text-only inputs to precisely controlled generation driven by multi-source heterogeneous sensor signals (e.g., audio, 3D, and physiological data). This paper presents a systematic review of cross-modal mapping and controllable generation under multi-source collaboration. More precisely, we propose a unified “cross-modal mapping and injection” taxonomy by abstracting the intervention logic of heterogeneous signals. Fundamentally, we analyze these mechanisms in a backbone-agnostic manner, delineating the architectural transition from legacy U-Net dependencies to scalable architectures like Diffusion Transformers (DiTs) and tracing the technical evolution from single-source atomic driving to complex multi-source collaborative paradigms. Our mechanistic analysis reveals that seamless feature fusion heavily relies on gradient conflict resolution, rigorous arbitration, and dynamic disentanglement under multi-constraint scenarios. Furthermore, by systematizing current evaluation metrics, we identify intrinsic quality-controllability trade-offs through performance game analysis (e.g., Pareto optimization), yielding a scientifically grounded technical selection guide. The study concludes that overcoming current generation limitations necessitates integrating Hardware-in-the-Loop (HIL) deployment, PDE-driven physical constraints, and causal inference, laying the foundation for next-generation robust and real-time generative models.

## 1. Introduction

In the evolutionary trajectory of Artificial Intelligence Generated Content (AIGC), Diffusion Models (DMs) have established themselves as the cornerstone of image generation by virtue of their exceptional capacity to fit high-dimensional data distributions [1]. The current paradigm is undergoing a fundamental transition—from single-modality text-driven generation (Text-to-Image, T2I), encompassing both high-resolution U-Net refinements [2] and scalable transformer paradigms [3,4,5], toward generation driven by non-visual, multimodal heterogeneous signals—with the aim of leveraging one-dimensional sequential audio, non-Euclidean topological 3D data, and high-dimensional sparse physiological signals to achieve fine-grained manipulation of the visual manifold [6,7]. However, this progression is impeded by deep-seated theoretical bottlenecks: heterogeneous signals and two-dimensional images exhibit a pronounced modality gap across representational dimensionality [8], spatiotemporal distribution [9], and geometric structure [10]. How to preserve representational topology invariance and suppress manifold distortion throughout the mapping and injection process has become a core challenge of pressing urgency in the field of controllable image generation [11].

In response to the aforementioned challenges, this paper conducts an in-depth deconstruction of prevailing literature and cutting-edge technologies, from which a unified cross-modal mapping and injection framework is distilled. This framework seeks to unpack, at the level of underlying mechanisms, the intervention logic through which heterogeneous signals perturb the generation process. Oriented toward the technical endpoints of distinct modalities, the framework establishes three core driving paradigms: first, sequential collaborative generation centered on the audio modality, which prioritizes instantaneous alignment of dynamic manifolds [12]; second, spatially consistent generation centered on 3D geometry, which emphasizes the robust injection of topological structures [6]; and third, semantic reconstruction generation centered on brain–computer interface (BCI) signals, which focuses on the decoding of latent priors and semantic restoration [13]. This taxonomic synthesis not only reveals the intrinsic unity underlying the injection of heterogeneous signals into generative models, but also establishes a representational baseline for the subsequent development of complex collaborative control.

As application scenarios grow increasingly complex, single-modality control is irreversibly evolving toward multi-source heterogeneous synergy [14]. Beyond fundamental theoretical research, this collaborative generation paradigm is profoundly reshaping diverse, complex downstream industrial applications. The demand for precise, multi-conditional synthesis has become critical in fields such as medical imaging analysis (where comprehensive frameworks [15,16], geometric masks [17], and pathological synthesis [18,19] jointly guide generation), autonomous driving simulation [20] (which requires strict topological alignment among bird’s-eye view layouts [21], panoramic depth maps [22], and dynamic text conditions [23,24]), virtual reality and digital human rendering (relying on the synchronization of audio streams [25,26,27,28] and 3D facial/skeletal priors [29,30]), and e-commerce virtual try-on systems (demanding rigorous identity preservation [31,32] alongside structural garment control [33,34] and manipulable wearing styles [35]). The immense industrial value of these applications further exacerbates the urgency of resolving the interference and gradient conflicts inherent in heterogeneous control flows. Under conditions of intertwined multi-dimensional constraints, distinct mapping operators and injection operators exhibit pronounced “gradient competition” within the optimization space, accompanied by severe “semantic-spatial conflict” [36]. Furthermore, to address the measurement challenge of collaborative efficacy, this paper constructs a multi-dimensional evaluation metric system oriented toward mapping fidelity and collaborative injection efficacy. Through performance analysis of different evolutionary pathways along the axes of controllable precision and generation quality, this paper distills a scientifically grounded technical selection guide, intended to serve as a decision-making reference for industrial tasks of varying complexity.

To explicitly distinguish the theoretical contributions of this review from the systematic summary of existing literature, the central novelties of this paper are outlined as follows:

(1) A Unified Mapping and Injection Taxonomy: Diverging from traditional surveys that predominantly enumerate empirical applications, this paper abstracts a backbone-agnostic cross-modal mapping taxonomy (Section 2). This framework systematically decouples legacy U-Net dependencies and exhibits high adaptability to the scalable architecture of Diffusion Transformers (DiTs).
(2) An Original Hierarchical Fusion Framework and Conflict Taxonomy: Moving beyond conventional literature summarization, an original “Representation-Interaction-Arbitration” hierarchical fusion framework is proposed in this review (Section 3.1). Concurrently, a novel conflict taxonomy is also proposed in this review (Section 3.4.2) to mathematically categorize latent gradient collisions and spatial-semantic antagonisms inherent in multi-source generation.
(3) A Novel Quantitative Evaluation Protocol (OMNP): To resolve the incommensurability of heterogeneous metrics across diverse research domains, the Ordinal Mapping Normalization Protocol (OMNP) proposed in this review is introduced (Section 4.2.1). This quantitative protocol projects discrete literature benchmarks into a unified, actionable Pareto coordinate system, facilitating explicit evaluations of quality-controllability trade-offs.

The remainder of this paper is organized as follows: Section 2 provides an in-depth analysis of the underlying mechanisms of cross-modal mapping and injection, examining the technical pathways of audio-temporal synchronization, 3D spatial consistency, and BCI-based semantic reconstruction respectively; Section 3 focuses on conflict resolution strategies and dynamic fusion schemes within multi-source heterogeneous collaboration; Section 4 systematically constructs a performance evaluation framework oriented toward mapping injection and collaborative consistency, and derives a technical selection guide tailored to different application scenarios; Section 5 concludes with a prospective discussion of open problems and future directions in this field.

## 2. Controllable Image Generation via Cross-Modal Mapping and Injection

Before analyzing modality-specific conditional injections, it is imperative to establish the theoretical evolution [37,38] of the foundational denoising engines. The generation paradigm has experienced a structural shift from the foundational Denoising Diffusion Probabilistic Models (DDPM) [1] and their accelerated implicit sampling variants (DDIM) [39] to the highly efficient Latent Diffusion Models (LDM) [40]. By projecting the optimization objective from the high-dimensional pixel space into a compressed latent manifold, LDM fundamentally unlocked the feasibility of high-resolution generation and laid the architectural groundwork for subsequent cross-modal interventions. Recently, the field is undergoing another paradigm leap with the emergence of Diffusion Transformers (DiT) [3], which replaces the traditional U-Net backbone with scalable transformer architectures, thereby providing a more native and isotropic substrate for multi-source tokenized control.

Building upon these robust foundational architectures and their powerful text-driven generative capabilities, incorporating non-visual modalities—such as audio, 3D geometry, and physiological signals—as control sources has become pivotal to achieving more immersive and personalized generation. Unlike conventional visual priors, these modalities exhibit pronounced representational heterogeneity. Centered on the overarching challenge of bridging the modality gap, this chapter first outlines a general framework for cross-modal mapping and elucidates the pathways through which heterogeneous signals are transformed into robust control flows based on differences in their physical properties and control characteristics. The chapter proceeds to examine audio temporal mapping, 3D geometry projection consistency, and physiological signal latent alignment [41], thereby establishing a representational foundation for the exploration of multi-source collaborative generation in subsequent chapters.

### 2.1. Cross-Modal Mapping and Injection Framework

At its essence, cross-modal mapping seeks to identify robust projections of non-visual modalities within a pre-trained two-dimensional generative space. With respect to the physical characteristics of different modalities, the research community has converged on two core alignment paradigms: first, “implicit semantic alignment” for audio and physiological signal [42,43], which aims to transform them into continuous prompt embeddings interpretable by the generative model through manifold alignment; second, “spatial consistency” control for 3D geometric modalities, which leverages three-dimensional structural priors to address the Janus problem and resolve spatial uncertainty in two-dimensional generation [44,45].

**Figure 1:**
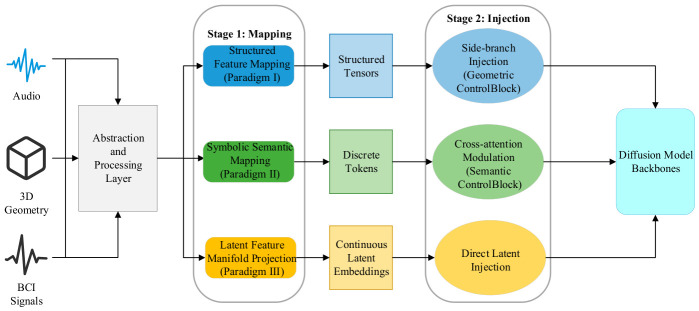
Schematic diagram of the cross-modal mapping and injection framework proposed in this paper.

To clarify the logical pathway through which heterogeneous modalities are injected into the generative space, this section synthesizes existing state-of-the-art work and distills the unified cross-modal mapping and injection theoretical framework illustrated in Figure 1. This framework reveals that the control mechanisms of heterogeneous modalities universally conform to an implicit two-stage paradigm: first, modality-specific encoding is applied to achieve normalized mapping within the feature space; subsequently, a standardized interface is employed to enable unified injection.

Stage 1: Normalized Mapping of Modality Features. Based on differences in signal physical properties and semantic hierarchy, existing technical approaches can be summarized as three parallel meta-paradigms:

(1) Paradigm I—Structured Mapping in Feature Space: Applicable to modalities with explicit physical structure or spatial topology, such as 3D geometry and audio time-frequency spectrograms. The core mechanism projects one-dimensional or three-dimensional signals into two-dimensional or three-dimensional feature tensors with spatial dimensions via a structured mapper [46,47], preserving the physical topological information of the original signal by lifting geometric features [48,49] and adapting it to the spatial inductive bias of convolutional neural networks or the isotropic token space of Vision Transformers through instance and layout projections [50,51,52].
(2) Paradigm II—Symbolic Representation in Semantic Space: Applicable to modalities with well-defined semantics but lacking spatial correspondence, such as audio classification labels and brain-decoded semantics. The core mechanism employs semantic decoders—such as visual instruction tuning models [53,54] and advanced tokenizers [55]—to discretize continuous signals into symbolic sequences [56] or text-guided instructions [57], establishing associations between non-visual signals and textual semantics through symbolic intermediaries [58]. In more recent developments, agents built upon multimodal large language models (MLLMs) have further enhanced the depth of symbolic semantic parsing and the capacity for adaptive alignment [59].
(3) Paradigm III—Latent Feature Distribution Alignment: Representing an end-to-end deep alignment strategy, this paradigm is applicable to high-dimensional abstract signals that are difficult to symbolize explicitly, such as latent Electroencephalography (EEG) and Functional Magnetic Resonance Imaging (fMRI) intent [60,61] and audio-derived emotion [62,63,64]. The core mechanism achieves direct alignment between non-visual manifolds and visual semantic manifolds through a learned alignment projector [65]. By deeply integrating mapping operators into the latent space of the DiT architecture, this pathway has achieved millisecond-level real-time cross-modal generation with high-fidelity alignment.

Stage 2: Unified Injection Pathways for Heterogeneous Features. Upon completion of representational mapping, heterogeneous modalities converge toward a common destination, interfacing with the generative backbone through three categories of standardized interfaces validated in this chapter:

(1) Side-Branch Spatial Stream Injection (based on Paradigm I): Structured feature tensors are directly merged into the intermediate feature representations (e.g., convolutional feature streams or patchified hidden states) of the denoising backbone via side-branch injection interfaces—such as ControlNet residual layers—imposing strong constraints on the geometric structure and layout of the generated image [46,50]. For high-resolution requirements, ControlNet++ substantially improves injection fidelity through a discriminative feedback mechanism [66].
(2) Cross-Attention Semantic Modulation (based on Paradigm II): Discrete symbolic sequences or continuous latent embeddings are injected as key-value pairs via cross-attention mechanisms [67,68,69], governing the categorical attributes [70] and stylistic characteristics [71,72] of the generated content. Recent advances introduce a decoupled dual-stream injection strategy that substantially mitigates gradient competition among multi-source heterogeneous semantics within the shared latent space [73,74].
(3) Direct Latent Injection and Hard Constraint Enhancement (based on Paradigm III): For continuous latent embeddings, this approach directly integrates the aligned features into the generative space. Serving as an efficacy-enhancing mechanism, it directly intervenes in the computation of attention maps or latent representations—through the application of geometric latent masking [75,76], correspondence-aware epipolar geometric constraints [77], or explicit semantic correspondence restrictions [78,79]—to forcibly embed physical priors into the generation pipeline in the form of hard biases.

### 2.2. Audio Modality Mapping and Injection for Temporally Synchronized Image Generation

Audio is a quintessentially non-stationary sequential signal that exhibits pronounced natural heterogeneity with respect to static visual modalities in terms of information density, temporal structure, and semantic abstraction hierarchy. How to precisely map one-dimensional audio waveforms and inject them into the two-dimensional image generation process remains one of the most challenging problems in the field of multimodal generation.

A consensus has emerged in the research community that, based on the representational form of audio features, the degree of intervention applied to the diffusion U-Net backbone, and the physical pathway of conditional injection, existing audio-conditioned image generation methods can be rigorously categorized into three mutually orthogonal and mutually exclusive mainstream paradigms: (1) Paradigm I: structured token mapping with decoupled parallel injection; (2) Paradigm II: pseudo-text token mapping with non-invasive injection; and (3) Paradigm III: native sequential token mapping with bypass modulation injection.

#### 2.2.1. Audio-Driven Image Generation via Structured Token Mapping and Decoupled Injection

This decoupled injection mechanism adheres to a “divide and conquer” design philosophy, premised on the view that audio and text belong to highly heterogeneous semantic spaces, and that forcing them to share a single conditional injection channel readily induces inter-modal interference. Accordingly, this paradigm explicitly extends the architecture of the pre-trained generative backbone to establish a physically isolated injection pathway for audio conditioning, thereby enabling precise disentanglement of semantic content from acoustic attributes.

**Figure 2:**
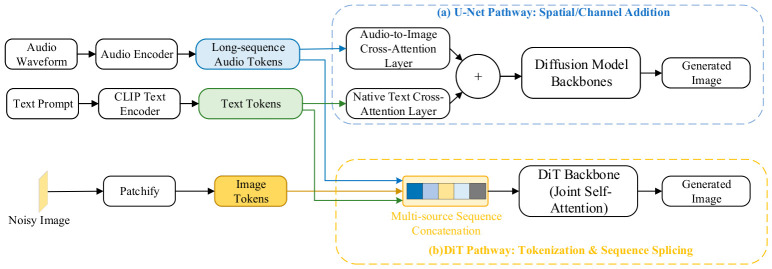
Schematic of structured token mapping and decoupled injection mechanism. (**a**) Parallel routing adaptation in classical convolution-based backbones; (**b**) Unified sequence concatenation in scalable transformer-based architectures.

As illustrated in Figure 2, the audio waveform is first mapped by a dedicated encoder into a sequential token sequence, aligning its representational shape with text embeddings. However, the specific injection routing inherently depends on the underlying generative backbone. Within classical U-Net frameworks, audio and text conditions are processed through parallel cross-attention layers, where intermediate image features act as shared queries to independently retrieve semantic and rhythmic attributes before being aggregated via gated fusion or residual summation. Conversely, as architectures scale toward Diffusion Transformers (DiTs), this parallel routing is superseded by a unified sequence concatenation strategy. Extracted audio, text, and patchified image tokens are directly flattened into a single 1D context sequence and fed into global self-attention blocks, enabling native cross-modal interaction within a shared manifold.

Despite these topological differences, both architectural adaptations successfully preserve pre-trained generative priors while achieving joint fine-grained control. For instance, SonicDiffusion [42] was the first to validate the viability of this paradigm within the U-Net architecture, successfully achieving disentangled control over audio style and textual content. MACS [80] further introduced multi-scale temporal aggregation to mitigate the attention dilution problem in long sequences, improving generation quality in complex acoustic scenarios.

Furthermore, the DiT pathway inherently blurs the task boundary between static image and temporal video generation. Because Transformer backbones flatten spatial latents into generic 1D sequences, emerging multimodal foundation architectures like UniForm [81] natively generalize the audio-driven image generation task into continuous temporal generation. By explicitly concatenating auditory and visual latents, this unified sequence concatenation paradigm treats static images simply as single-frame sequences, allowing acoustic and semantic features to interact via global self-attention for unprecedented cross-modal temporal alignment. In summary, Paradigm I offers the highest degree of temporal fidelity and modality disentanglement among the three audio-driven paradigms.

#### 2.2.2. Audio-Driven Image Generation via Pseudo-Text Token Mapping and Non-Invasive Injection

This injection mechanism strictly adheres to the principle of “zero structural perturbation,” aiming to achieve unobtrusive conditional injection by highly compressing audio conditions and mapping them directly into pseudo-text tokens consumable by the text pathway—all while keeping the main architecture of the pre-trained diffusion model entirely frozen.

**Figure 3:**
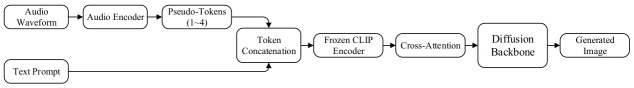
Schematic of the pseudo-text token mapping and non-invasive injection mechanism.

As illustrated in Figure 3, the core of this paradigm lies in the extreme compression mapping from audio to text tokens. The raw audio waveform is first processed by a lightweight acoustic encoder to extract global features, which are then compressed into merely 1–4 learnable pseudo-text tokens via a Textual Inversion-style projector. These tokens are explicitly constrained to reside within the pre-trained CLIP text embedding space, achieving full alignment with ordinary text tokens in both dimensionality and distribution.

During the conditional injection stage, the generated pseudo-text tokens are inserted directly into the user’s natural language prompt via an early sequence concatenation strategy, forming a hybrid token sequence of length 77 + N. This sequence is fed in its entirety into the frozen CLIP text encoder, and the extracted embedding vectors are subsequently processed through the backbone’s native text cross-attention mechanism without modification. Since the backbone network has no means of detecting the true modality origin of the tokens, audio conditions are treated entirely as ordinary text tokens.

AudioToken [58], GlueGen [82], and AAI [83] collectively established this technical paradigm. During training, this approach requires the optimization of only a handful of token embeddings and incurs zero additional computational or memory overhead at inference time. However, the lossy compression from continuous waveforms to merely a few tokens inevitably results in severe loss of fine-grained temporal detail—such as transient rhythms and dynamic pitch—while pseudo-text tokens may also introduce subtle semantic drift through attention competition with genuine text tokens. In summary, the audio-to-text token mapping mechanism achieves plug-and-play audio conditioning at minimal engineering cost, yet it is subject to inherent bottlenecks in temporal fidelity and control granularity.

#### 2.2.3. Audio-Driven Image Generation via Native Sequential Token Mapping and Bypass Modulation Injection

This mechanism represents the most recent technical advancement in the field as of 2025, with the core objective of systematically overcoming the inter-modal semantic interference and fine-grained temporal information loss that are prevalent across the preceding two paradigms.

**Figure 4:**
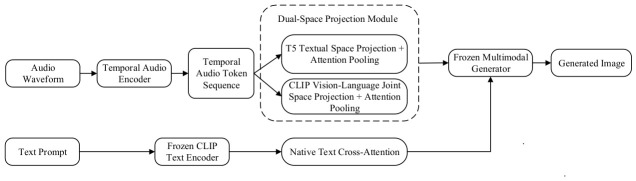
Schematic of native temporal token mapping and bypass modulation injection mechanism.

As illustrated in Figure 4, this paradigm entirely abandons the conventional approaches of forcing audio into fixed-length alignment (Paradigm I) or compressing it into pseudo-text tokens (Paradigm II). Instead, the raw audio waveform is directly converted by a temporally dedicated encoder into a variable-length native sequential token sequence, fully preserving dynamic details and non-stationary characteristics. More critically, this paradigm enforces strict zero structural modification to the pre-trained diffusion backbone: the audio conditioning stream bypasses the native text cross-attention pathway entirely.

With regard to the conditional injection mechanism, Paradigm III introduces a dual-space independent projection and bypass feature modulation strategy. Specifically, the sequential token sequence first enters a lightweight side network comprising two parallel projection heads, which align audio features to the T5 text semantic space and the CLIP vision-language joint space, augmented by attention pooling to enable comprehensive integration of global and local information. The projected audio representations are then injected directly into the intermediate feature representations of the backbone (accommodating both multi-scale convolutional maps and DiT patch tokens) in the form of feature modulation—such as AdaGN, FiLM, or residual summation (as indicated by the injection pathway from the Dual-Space Projection Module to the Frozen Multimodal Generator in Figure 4)—rather than being routed through the cross-attention module. This complete physical separation of pathways ensures full disentanglement between text semantic control and audio temporal control.

SeeingSounds [84], as the current state-of-the-art representative of this paradigm, was the first to demonstrate the significant advantages of pure alignment independent conditioning on long-duration complex acoustic events. The visual content it generates surpasses both preceding paradigms in terms of temporal synchronization, dynamic consistency, and text fidelity, fully embodying the fundamental breakthrough this approach achieves in modality disentanglement and temporal fidelity. In summary, Paradigm III-through zero structural invasion, dual-space precise alignment, and bypass modulation-is the first to simultaneously achieve high text control precision and fine-grained audio temporal detail.

#### 2.2.4. Analysis of Temporally Synchronized Image Generation via Audio Modality Mapping and Injection

**Figure 5:**
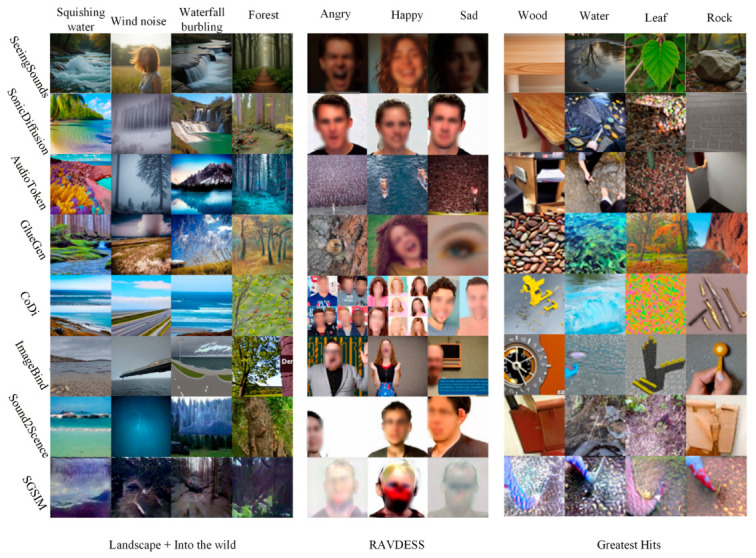
Qualitative comparison of temporally coordinated image generation under various audio-modality mappings and injection schemes across RAVDESS, Landscape, and Greatest Hits benchmarks. Images adapted from the supplementary files of ref. [84] to incorporate comparative performance benchmarks.

As illustrated in Figure 5, the three paradigms exhibit pronounced visual feature differentiation across distinct semantic dimensions. Paradigm I (SonicDiffusion), leveraging its explicit conditional injection mechanism, exhibits exceptionally strong transient acoustic responsiveness and renders sharp textural edges in specific applications, particularly when evaluated on the combined Landscape [85] + Into the Wild [86] dataset. In contrast, Paradigm II (AudioToken/GlueGen), based on pseudo-text mapping, maintains compositional robustness but exhibits a marked tendency toward semantic smoothing when handling the non-rigid emotional nuances of RAVDESS [87]. Paradigm III (SeeingSounds), leveraging its dual-space independent projection mechanism, achieves a decisive lead in complex physical interaction consistency (Greatest Hits [88]) and deep semantic alignment, thereby resolving the semantic occlusion problem that arises between heterogeneous modalities.

In summary, the underlying architecture of the mapping paradigm directly determines both the controllable precision and the generalization boundary of the generated content: Paradigm I prioritizes the visual replication of acoustic physical attributes; Paradigm II achieves lightweight alignment at the semantic level; while Paradigm III represents the current technical ceiling for handling complex cross-modal conflicts and achieving full-scenario generalization.

### 2.3. 3D Geometry Modality Mapping and Injection for Spatially Consistent Image Generation

Three-dimensional geometric modalities are characterized by intrinsic properties including high-dimensional spatial topology, multi-view geometric constraints, and explicit physical attributes. Unlike audio or physiological signals, which rely primarily on implicit semantic alignment, the core motivation for incorporating 3D priors is to address the spatial uncertainty inherent to 2D diffusion models—that is, when generating multiple viewpoints of the same object, such models frequently exhibit structural drift, texture flickering, or the Janus problem (e.g., the back of an object erroneously displaying frontal facial features) due to the absence of three-dimensional physical constraints. It should be emphasized that this section does not concern traditional 3D reconstruction; rather, it focuses on how 3D priors can be leveraged to constrain the 2D image generation process, spanning from multi-view synthesis to high-fidelity generation with intrinsic consistency. Based on the depth of intervention that geometric conditioning exerts on the generative manifold, existing methods can be organized into four paradigms: from explicit geometry map mapping with zero-convolution injection, to parametric pose mapping with decoupled attention injection, to multi-view feature mapping with geometric bias injection, and finally to intrinsic 3D mapping with rendering projection injection.

#### 2.3.1. Spatially Consistent Image Generation via Explicit Geometry Map Mapping and Zero-Initialized Injection

The explicit geometry cross-domain injection mechanism adheres to a “geometric determinism first” design philosophy. To address the problem of uncontrollable topological structure in pure text-driven generation, this mechanism introduces 2.5D intermediate representations with well-defined physical meaning—such as normal maps or depth maps—as strong geometric constraints. By explicitly injecting these spatial cues into the denoising backbone, the method aims to enforce strict adherence of the generated 2D texture to a predefined geometric skeleton, thereby eliminating the geometric drift commonly observed in multi-view generation.

**Figure 6:**
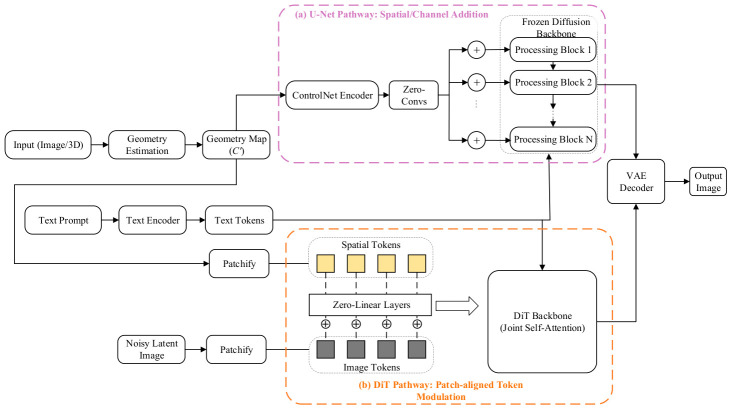
Schematic of explicit geometry map mapping and zero-initialized injection mechanism. (**a**) Spatial feature modulation in classical convolution-based backbones; (**b**) Tokenized sequence modulation in scalable transformer-based architectures.

As illustrated in Figure 6, the core architecture comprises a shared geometric mapping phase followed by a bifurcated, backbone-specific injection phase based on zero-initialization strategies:

(1) Shared Geometric Feature Mapping: The input data (Image/3D) is first processed by a geometry estimation module and mapped into a geometry map ($C^{\prime }$) strictly aligned with the target image space. This step transforms implicit three-dimensional structures into explicit two-dimensional tensors, resolving the representational heterogeneity between 3D geometry and 2D images in the feature space.
(2) Bifurcated Zero-Initialized Injection: To preserve the latent distribution of the pre-trained text generation model, the extracted geometric constraints are injected using weight-zeroed layers, effectively preventing severe gradient perturbations at the onset of training. The injection routing diverges based on the underlying architecture:
  **(a)**  U-Net Pathway (Spatial/Channel Addition): Following the classic ControlNet paradigm, a trainable bypass encoder replicates the U-Net blocks to extract multi-scale geometric features. These features pass through Zero-Convs (zero-initialized convolutions) and are directly added (⊕) to the spatial feature maps of the Frozen Diffusion Backbone via residual connections. Text tokens are injected independently via cross-attention.
  **(b)**  DiT Pathway (Patch-aligned Token Modulation): For Diffusion Transformers, the paradigm abandons convolutional bypass networks. Instead, both the geometry map ($C^{\prime }$) and the noisy latent image are patchified into sequence tokens. To preserve strict topological isomorphism, the Spatial Tokens and Image Tokens maintain a 1-to-1 spatial alignment. The spatial tokens are processed through Zero-Linear Layers and fused with the image tokens via element-wise addition (⊕). The geometrically modulated tokens, alongside the globally encoded text tokens, are subsequently fed into the Joint Self-Attention blocks of the DiT backbone.

This structured approach has been validated across multiple state-of-the-art models. Wonder3D [89] employs a cross-domain attention mechanism that, while incorporating improvements over standard ControlNet, remains consistent in its core philosophy—explicitly leveraging estimated normal maps as strong geometric constraints to ensure high consistency between surface details and overall geometric topology when generating back-view images. MVPaint [90] directly uses depth maps and normal maps rendered from untextured meshes as conditional inputs during texture generation, producing high-frequency textures in UV space that precisely match the geometric structure through a bypass injection mechanism, fundamentally eliminating ghosting and misalignment artifacts. Garment3DGen [91], targeting garment generation, leverages human pose maps and depth maps to precisely control wrinkles and deformations, achieving high-fidelity control over non-rigid objects.

Extending this structural exactness to the DiT pathway illustrated in Figure 6b, recent cutting-edge foundation architectures such as RelaCtrl [92] and OminiControl [73] have successfully ported explicit spatial modulation to highly scalable Transformers. Rather than relying on heavy convolutional bypasses, these frameworks patchify spatial geometry maps into geometrically aligned tokens. By passing these representations through zero-initialized linear layers and fusing them with image tokens at targeted Transformer blocks, they secure rigorous structural determinism with minimal additional parameter overhead, demonstrating the universal applicability of patch-aligned modulation.

In summary, the explicit geometry map cross-domain injection mechanism—by introducing physically meaningful 2.5D map representations—trades increased inference computational overhead for a high degree of controllability and determinism in the geometric structure of generated outputs. It currently stands as the mainstream solution for high-precision industrial-grade reconstruction and texture generation tasks.

#### 2.3.2. Spatially Consistent Image Generation via Parametric Pose Mapping and Decoupled Attention Injection

Unlike explicit injection, which prioritizes pixel-level geometric alignment, the parametric pose mapping mechanism adopts a “viewpoint-semantic disentanglement” strategy. This approach treats three-dimensional viewpoint variation as a special form of semantic instruction, endowing the diffusion model with “viewpoint-awareness” by mapping camera extrinsic parameters into high-dimensional embedding vectors that are isomorphic with text representations. This design aims to enable the model to “learn” how to distribute semantic content correctly in accordance with viewpoint logic—for instance, automatically suppressing facial features when generating back-view images—thereby achieving semantic consistency in multi-view synthesis within the implicit feature space.

**Figure 7:**
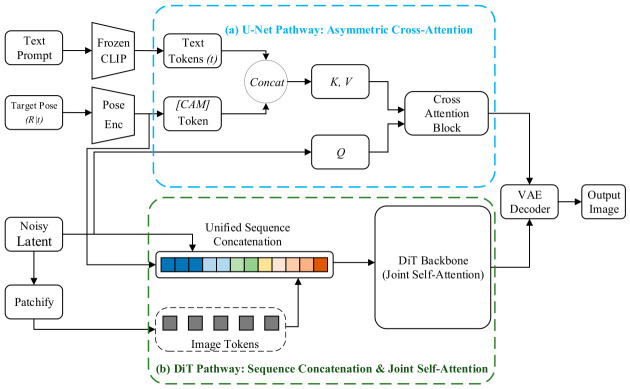
Schematic of parametric pose mapping and decoupled attention injection mechanism. (**a**) Asymmetric cross-attention injection in classical convolution-based backbones; (**b**) Joint self-attention integration in scalable transformer-based architectures.

As illustrated in Figure 7, the core architecture comprises a shared parametric encoding phase followed by bifurcated, backbone-specific attention injection pathways:

**(1)**  Shared Parametric Pose Mapping: The input target camera pose (R|t) is flattened and projected into a high-dimensional feature space via a multi-layer perceptron (Pose Enc), forming a [*CAM*] token. Simultaneously, the Text Prompt is encoded by a frozen CLIP model into Text Tokens (*t*). This shared front-end accurately realizes the mapping from physical geometric parameters to implicit semantic symbols, aligning them with textual instructions in both dimensionality and distribution.
**(2)**  Bifurcated Decoupled Attention Injection: The mechanism fuses these multimodal tokens into the generative backbone using decoupled pathways, preventing conditional parameters from prematurely interfering with spatial features:
  (a) U-Net Pathway (Asymmetric Cross-Attention): In standard convolutional architectures, the injection relies on an asymmetric cross-attention flow. The Text Tokens and [*CAM*] Token are concatenated along the sequence dimension to construct the joint Key and Value pairs (*K*, *V*). Meanwhile, the Noisy Latent variable is linearly projected to exclusively provide the Query vector (*Q*). Through this Cross-Attention Block, the spatial image features dynamically retrieve content and viewpoint semantics without altering their own structural topology.
  (b) DiT Pathway (Sequence Concatenation & Joint Self-Attention): For Transformer-based backbones, the asymmetric *Q-K/V* distinction is explicitly abandoned. The Noisy Latent is first processed through a Patchify module to yield Image Tokens. Subsequently, the Image Tokens, Text Tokens, and the [*CAM*] Token are all directly merged into a single Unified Sequence Concatenation. This extensive 1D sequence is then fed directly into the DiT Backbone, where decoupled multimodal tokens interact natively and bidirectionally via global Joint Self-Attention.

This approach has been validated across several representative works. Zero-1-to-3 [93] was the first to encode camera viewpoint differences as embedding vectors and concatenate them with image features, successfully endowing 2D diffusion models with novel view synthesis capability and demonstrating the feasibility of implicit pose control. MVDream [94] extended this mechanism to the text-to-3D domain, fine-tuning on large-scale 3D datasets to enable camera tokens to precisely govern global 3D structure, substantially mitigating the geometric inconsistency inherent in purely text-driven generation. MV-Adapter [95] employs a lightweight adapter module that achieves multi-view generation capability solely through camera token concatenation, without modifying the original weights of the pre-trained model; its decoupled attention mechanism ensures that camera conditioning does not interfere with textual semantic expression, striking a balance between high fidelity and high consistency.

Representing the Transformer-based trajectory illustrated in Figure 7b, recent foundation frameworks such as Hunyuan3D-1.0 [96] integrate Multi-View Diffusion Transformer (MV-DiT) modules that inherently validate this sequence-level concatenation paradigm. Rather than relying on asymmetric cross-attention for novel view synthesis, MV-DiT parameterizes target camera poses into specific embeddings that are directly concatenated with multi-view image latent codes and text tokens. This unified 1D sequence allows joint self-attention to elegantly resolve complex multi-view semantic disentanglement entirely within the implicit 2D space, providing highly consistent multi-view images prior to any explicit 3D reconstruction.

In summary, the camera pose encoding and decoupling mechanism achieves flexible control over the generative viewpoint of diffusion models with minimal architectural modification. While it falls short of explicit injection paradigms in terms of pixel-level geometric alignment, its strong semantic generalization capability makes it an important technical pathway for current text-driven 3D generation.

#### 2.3.3. Spatially Consistent Image Generation via Multi-View Feature Mapping and Geometric Bias Injection

Unlike Paradigm II, which broadcasts camera pose solely as a global semantic token, the geometry-aware attention mechanism adopts a philosophy of “physically constrained geometric feature interaction.” This approach holds that the consistency of multi-view image generation fundamentally derives from the epipolar geometric relationships between pixels. Accordingly, this paradigm explicitly introduces epipolar or ray-based constraints by modifying the underlying attention operator, compelling the model to attend exclusively to feature regions that are physically correspondent. This “hard constraint” mechanism deterministically eliminates irrelevant feature interference, ensuring that every generated image is geometrically self-consistent.

**Figure 8:**
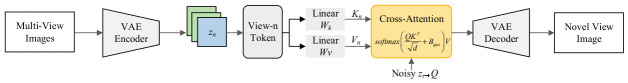
Schematic of multi-view feature mapping and geometric bias injection mechanism.

As illustrated in Figure 8, the core architecture of this approach is built upon a frozen generative backbone (transitioning from classical U-Net to scalable DiT), with the key modifications occurring within its internal cross-attention modules. The architecture primarily comprises two stages: multi-view latent feature extraction and geometry-biased attention computation.

(1) Multi-View Latent Feature Extraction and Key-Value Pair Construction: The input multi-view images or single-image conditions are first mapped by a frozen VAE encoder into a set of multi-view latent features *z*_1_, *z*_2_, …, *z_N_*. These features are subsequently linearly projected into sequences of key (K) and value (V) vectors. Unlike conventional approaches, these K vectors are not unordered image patches but rather feature carriers that encode the geometric information of specific viewpoints.
(2) Geometry-Biased Attention Computation: This constitutes the core innovation of the mechanism. During the denoising process of the denoising backbone, the noisy latent variable generates the query vector Q. Conventional cross-attention allows Q to interact with K vectors from all viewpoints indiscriminately, which readily leads to texture inconsistency. As shown in Figure 8, this method modifies the attention score computation formula:

(1) $$Attention(Q,K,V)=softmax(\frac{QK^{T}}{\sqrt{d}}+B_{geo})V$$

Here, *B_geo_* denotes the explicit geometric bias matrix. This matrix acts as an additive mask or weight bias, directly intervening in the computation of the attention map. It calculates geometric correspondence based on the pixel coordinates associated with Q and K—if two pixels do not satisfy a geometric correspondence relationship (e.g., they do not lie on the same epipolar line), *B_geo_* imposes a large negative value, thereby blocking the flow of invalid information.

Depending on how *B_geo_* is constructed, this approach gives rise to multiple technical pathways. InstantMesh [97] proposes an epipolar attention mechanism that constructs *B_geo_* as an epipolar mask based on the principles of epipolar geometry, reducing the two-dimensional full-image search to a one-dimensional epipolar line search—simultaneously lowering computational complexity and ensuring geometric consistency at the physical level, serving as a key enabler of second-level high-quality mesh reconstruction. Furthermore, SPAD [98] and ViewDiff [99] adopt a ray-conditioned strategy, directly injecting camera ray information—in the form of Plücker coordinates or ray directions—into *B_geo_* or the embeddings of the attention layers, enabling the model to perceive the spatial ray direction corresponding to each pixel and generate novel views that conform to the laws of perspective projection.

In summary, the geometry-aware attention mechanism transforms hard geometric constraints into soft attention biases by modifying the underlying feature interaction logic. Without requiring expensive volumetric rendering processes such as NeRF, this approach achieves consistency comparable to native 3D methods, making it a key solution for balancing generation quality, geometric precision, and inference speed.

#### 2.3.4. Spatially Consistent Image Generation via Intrinsic 3D Mapping and Rendering Projection Injection

Unlike the preceding three paradigms, which attempt to patch or constrain 3D consistency within the two-dimensional feature plane, the structured 3D latent representation injection mechanism adopts an “intrinsic consistency” generative philosophy, achieving a paradigm leap from “image correction” to “generation in elevated dimensions, observation in reduced dimensions.” This approach transfers the denoising process of the diffusion model into a structured 3D latent space—first generating a physically conserved 3D feature field, then projecting it back to the 2D plane via neural volume rendering. This “natively 3D” strategy fundamentally eliminates the geometric conflicts commonly encountered in traditional 2D generation, ensuring that the output image sequences maintain strict structural and textural consistency across arbitrary viewpoints.

**Figure 9:**
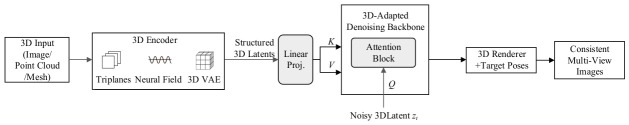
Schematic of intrinsic 3D mapping and rendering-projection-based injection mechanism.

As illustrated in Figure 9, the core architecture of this approach realizes the transition from “2D generation with post-processing” to “native 3D generation,” comprising three key stages: explicit 3D encoding, 3D latent space denoising, and neural volume rendering decoding.

(1) Explicit 3D Encoding and Spatial Dimensionality Elevation: The input modality—whether a single image, point cloud, or mesh—is first mapped by a dedicated 3D encoder into a compact and structured 3D representation. Unlike the 2.5D maps employed in Paradigm I, these representations typically take the form of tri-planes, neural fields, or 3D VAE latent variables. Rather than being viewpoint-dependent, these representations describe the geometric and textural distribution of an object as an objectively existing physical field.
(2) 3D Latent Space Denoising: As shown in the central region of the figure, this architecture abandons the conventional 2D VAE compression pathway. The foundational 2D denoising backbone is adapted to accommodate 3D data distributions. The query vector Q in the attention mechanism no longer derives from the noisy latent variable of a 2D image, but directly from the noisy 3D latent variable z_t (e.g., a noise-corrupted tri-plane feature map). External conditioning signals—such as text or image features—are injected directly into the 3D feature channels via cross-attention. This means the model no longer learns the arrangement of pixels, but rather the spatial distribution of voxels or tri-plane features.
(3) Neural Volume Rendering Decoding: Upon completion of denoising, the output is no longer decoded into an image via a 2D decoder; instead, it is fed directly into a 3D renderer—such as a NeRF-based volumetric renderer. In conjunction with the given target pose, the renderer generates multi-view images from the clean 3D latent representation through ray integral computation. Since all viewpoint images are projected from the same 3D model, they are mathematically guaranteed to satisfy strict multi-view consistency.

This approach has been validated across several representative works. GenVS [100] models the generation target as a 3D tri-plane distribution, achieving 360-degree generation without blind spots and completely eliminating the multi-face problem by inflating or adapting the convolutional kernels of the 2D diffusion model to process tri-plane features. Compress3D [101] introduces an efficient 3D VAE mechanism that substantially reduces the computational and memory overhead of 3D generation by training the diffusion model on a compressed latent space. TRELLIS [102] constructs a more refined structured latent representation that, while maintaining high geometric fidelity, significantly enhances the diversity and textural detail of generated content, demonstrating considerable potential for large-scale 3D asset generation.

In summary, the structured 3D latent representation injection mechanism—through its dimensionality-elevated generation strategy—trades partial loss of pre-trained 2D priors and increased training overhead for geometrically accurate consistency in the physical sense. This approach is particularly well-suited to industrial design and game asset production scenarios where strict structural topology requirements must be met.

#### 2.3.5. Analysis of Spatially Consistent Image Generation via 3D Geometry Modality Mapping and Injection

**Figure 10:**
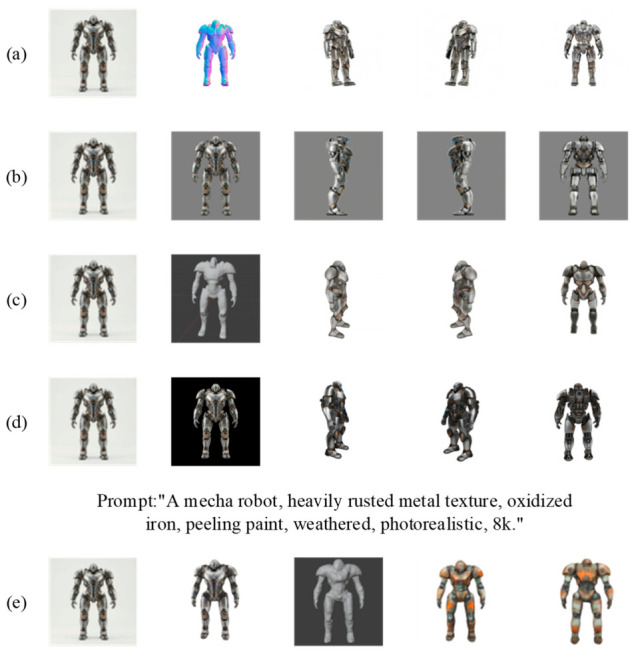
Qualitative comparison of spatially-consistent image generation and manipulation: (**a**) Paradigm I (Wonder3D) utilizing RGB-encoded surface normal maps; (**b**) Paradigm II (MV-Adapter) employing grayscale shaded meshes for geometric consistency; (**c**) Paradigm III (InstantMesh) featuring neutral gray untextured meshes; (**d**) Paradigm IV (TRELLIS) using high-contrast specular mesh previews for geometric alignment; (**e**) 3D-aware editing paradigm (Generic 3D Adapter) showcasing realistic iron oxide textures.

As illustrated in Figure 10, the four geometric conditioning paradigms exhibit pronounced functional differentiation in maintaining spatial consistency. Paradigm I (Wonder3D), shown in Figure 10a, leverages the explicit guidance of the normal map in the second column to ensure strict topological alignment between the textured meshes in the subsequent three columns and the input image. Paradigm II (MV-Adapter), shown in Figure 10b, demonstrates superior semantic inference capability when handling the back-view in the fifth column, ensuring cross-viewpoint lighting and shading consistency. Paradigm III (InstantMesh), shown in Figure 10c, validates through the untextured mesh in the second column the efficiency of epipolar constraints in constructing manifold-continuous surfaces. Paradigm IV, represented by TRELLIS and shown in Figure 10d, fundamentally eliminates inter-viewpoint geometric adhesion and texture flickering across the continuous rotational viewpoints in columns three through five, by virtue of structured latent space denoising.

Furthermore, the editing paradigm exemplified by the Generic 3D Adapter [103] demonstrates the potential of 3D-aware architectures for locally controllable manipulation. As shown in Figure 10e, the model achieves a seamless, continuous distribution of iron oxide texture across 3D space while preserving the original mech geometric structure intact. In summary, the underlying logic of each mapping paradigm determines its application boundary: Paradigm I prioritizes high-precision industrial reconstruction; Paradigms II and IV hold respective advantages in semantic generation and physical consistency; Paradigm III provides the efficiency support necessary for real-time interaction.

### 2.4. Semantic Reconstruction Image Generation for Brain–Computer Interface Signal Mapping and Injection

EEG and fMRI, as typical implicit physiological modalities, exhibit fundamental differences from static visual modalities in signal-to-noise ratio (SNR), spatiotemporal resolution, and semantic abstraction levels. Unlike audio signals with relatively consistent physical characteristics, neural signals face two fundamental challenges: low SNR coupled with semantic sparsity, as well as significant inter-individual variability. These challenges result in disparate neural response patterns evoked by the same visual stimulus across different individuals. Therefore, constructing a generalizable neural decoding mechanism that tolerates individual differences and bridges the gap from pixel-level “visual reconstruction” to feature-level “semantic alignment” represents a critical scientific question in neural decoding and multimodal generation.

Existing brain signal-conditioned image generation methods can be categorized into three mainstream paradigms based on neural feature decoding strategies and semantic alignment pathways: (1) latent prior retrieval mapping and aligned anchor injection; (2) cascade semantic injection via neural symbolic decoding mapping; and (3) direct semantic injection via latent feature embedding mapping. It is worth noting that due to the extreme scarcity of paired EEG/fMRI-image datasets, current brain-driven image generation predominantly relies on the cross-attention mechanisms of pre-trained U-Net LDMs (e.g., Stable Diffusion). However, the explicit semantic decoding and injection principles abstracted in this section offer valuable conceptual insights. As dataset scales grow, these injection paradigms have the potential to be adapted into scalable DiT architectures, following the evolutionary trajectory currently observed in audio and 3D modalities.

#### 2.4.1. Brain Signal-Driven Image Generation via Latent Prior Retrieval Mapping and Aligned Anchor Injection

The cross-modal brain-visual latent feature retrieval and injection mechanism follows the design principle of “visual prior reuse,” aiming to achieve high-fidelity “visual reconstruction.” This paradigm posits that neural signals inherently lack abundant visual physical details, and direct mapping tends to cause mode collapse. Consequently, this mechanism reframes the decoding task as a “feature anchoring” process: utilizing brain signals as indices to retrieve nearest neighbor prototypes within a pre-constructed visual latent manifold, and achieving pixel-level perceptual restoration through reusing these high-quality visual priors.

**Figure 11:**
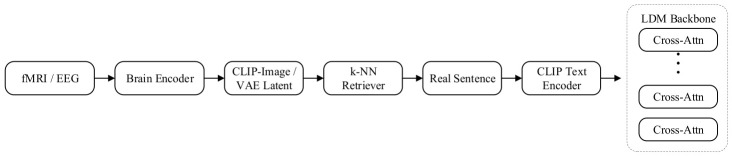
Schematic of latent prior retrieval mapping and aligned anchor injection mechanism.

As illustrated in Figure 11, this mechanism constructs an explicit processing pipeline encompassing feature alignment, nearest neighbor retrieval, and conditional injection, enabling precise anchoring from the neural manifold to the generative manifold. Neural-visual feature alignment first extracts spatiotemporal features from fMRI/EEG via dedicated encoders (e.g., masked autoencoders or 3D convolutional networks), and projects them onto a pre-trained CLIP image space or VAE latent space to achieve modality alignment, ensuring that heterogeneous bioelectric signals are geometrically distributed as consistently as possible with real visual stimulus features. The prior retrieval based on k-nearest neighbors (k-NN) constitutes the core distinguishing element of this paradigm from end-to-end generation: utilizing the aligned brain features as query vectors, k-NN retrieval is performed over large-scale image-text datasets (e.g., LAION) by computing cosine similarity in feature space to locate visual priors or semantic labels that best match the current brain activity pattern. In the semantic enhancement and conditional injection phase, the retrieved text descriptions are encoded via the CLIP text encoder and injected into the generation process through cross-attention layers in the diffusion model’s denoising network, leveraging the rich semantic information carried by the retrieved visual surrogates to guide generation, thereby markedly mitigating the semantic ambiguity of brain signals.

Representative works of this paradigm include Brain-Diffuser [104] and Mind-Video [105], which have respectively established performance benchmarks for the retrieval-alignment approach on static image and dynamic video reconstruction tasks. Brain-Diffuser employs an explicit two-stage retrieval strategy: in the semantic pathway, CLIP image features decoded from fMRI are used to retrieve nearest-neighbor text descriptions from datasets such as LAION-5B as semantic conditions; in the visual pathway, VDVAE latent variables are leveraged for low-level feature anchoring to achieve high-fidelity reconstruction. Mind-Video extends the retrieval-alignment concept to the temporal dimension, proposing a multimodal contrastive masked autoencoder that powerfully aligns brain signals to the CLIP visual space through contrastive learning on large-scale fMRI-video pairs. Its core contribution lies in the use of an implicit retrieval mechanism to successfully recover temporally coherent video streams from limited fMRI data, demonstrating that sufficient latent space alignment can enable a leap from sparse brain signals to continuous video frames.

In summary, the cross-modal brain-visual latent feature retrieval injection mechanism partially transforms the generation task into a retrieval task by introducing external memory, significantly reducing training difficulty and enabling the model to exhibit remarkable perceptual fidelity in dynamic visual reconstruction—though its semantic flexibility remains constrained by the capacity and coverage of the retrieval database.

#### 2.4.2. Brain Signal-Driven Image Generation via Neural Symbolic Decoding Mapping and Cascade Semantic Injection

The cross-modal brain-language explicit semantic bridging and injection mechanism adheres to the design principle of “semantic information bottleneck,” aiming to achieve interpretable “explicit semantic alignment.” Confronting high-dimensional noise in brain signals, this paradigm introduces natural language as a universal interface, leveraging large language models (LLMs) to transcode brain activity into discrete textual descriptions. This process serves as a semantic filter, directly stripping away physiological noise unrelated to visual content, and ensuring that the generation process adheres to strict logical semantics rather than pixel-level statistical regularities.

**Figure 12:**
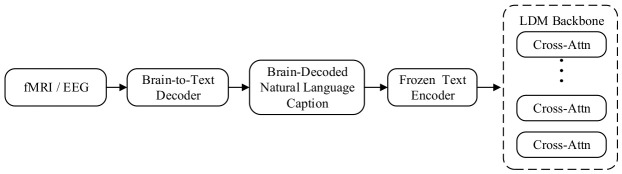
Schematic of neural symbolic decoding mapping and cascade semantic injection mechanism.

As illustrated in Figure 12, this mechanism constructs a typical cascade generation pathway, achieving cross-modal logical bridging through explicit semantic decoding and standard text-conditional injection. In the neural-symbolic semantic transcription phase, raw fMRI/EEG signals are first processed via a dedicated brain-to-text decoder, which typically comprises a pre-trained brain feature extractor and a large language model adapter, responsible for decoding continuous noisy neural activity patterns into discrete symbol sequences. This process serves as a semantic filter, directly stripping away physiological noise in brain signals unrelated to visual content while preserving only high-level semantic intent. Explicit semantic bridging is the signature feature of this paradigm: the decoder outputs human-readable, discrete natural language descriptions that constitute an explicit bridge connecting the brain and images, ensuring that the generation process adheres to strict semantic logic rather than pixel-level statistical regularities. In the standard text-conditional injection phase, natural language descriptions are converted into high-dimensional text embeddings via frozen text encoders (e.g., CLIP text encoder or T5), which are subsequently used as conditional signals to control the denoising trajectory through cross-attention layers in the diffusion model’s denoising network. By employing frozen off-the-shelf text encoders, this mechanism maximally reuses the semantic understanding and generative priors of pre-trained diffusion models.

Representative works of this paradigm include MindSemantix [106] and Semantic Prompts [107], which establish a new brain decoding pathway centered on language. MindSemantix proposes an end-to-end brain-language-vision framework that first leverages contrastive learning to pre-train a brain encoder aligned with the BLIP-2 text space, then fine-tunes a large language model to generate high-quality image descriptions containing object categories, attributes, and relationships from fMRI. These brain-decoded descriptions are directly fed into the pre-trained generative backbone (e.g., Stable Diffusion) for image generation. This cascade strategy enables MindSemantix to significantly outperform traditional methods on semantic accuracy metrics (e.g., CLIP score) and generate logically coherent complex scenes. Semantic Prompts addresses the low signal-to-noise ratio characteristic of EEG signals by introducing a multi-level semantic alignment strategy, training models to predict corresponding semantic prompts from EEG to guide the diffusion model, effectively circumventing the spatial information loss problem in EEG signals, and achieving high-quality category-level reconstruction by supplementing visual details through language priors.

In summary, the cross-modal brain-language explicit semantic bridging and injection mechanism transforms the incomprehensible neural decoding problem into an interpretable text generation problem by introducing a discrete symbolic system as an intermediary. Although this design sacrifices pixel-level spatial correspondence, it substantially enhances the semantic accuracy and logical coherence of generated content, making it particularly well-suited for mind-reading and dream-reconstruction tasks involving complex semantic understanding.

#### 2.4.3. Brain Signal-Driven Image Generation via Latent Feature Embedding Mapping and Direct Semantic Injection

The cross-modal brain-text latent embedding mapping and injection mechanism adheres to the design principle of “latent feature distribution alignment,” aiming to achieve end-to-end “implicit semantic alignment.” In response to potential information loss from explicit text decoding, this paradigm learns a nonlinear projection function to directly align the neural manifold to the continuous vector space of pre-trained text encoders (e.g., CLIP). The mapped brain features constitute machine-interpretable “continuous prompt embeddings,” thereby directly driving the diffusion model to generate images conforming to the brain’s latent intent without requiring explicit linguistic intermediaries.

**Figure 13:**
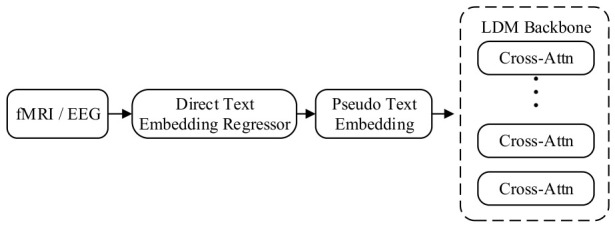
Schematic of latent feature embedding mapping and direct semantic injection mechanism.

As illustrated in Figure 13, this mechanism eschews explicit text decoding steps and constructs a direct projection pathway from neural signals to the semantic space of generative models. In the direct text embedding regression phase, raw fMRI/EEG signals are processed via a dedicated direct text embedding regressor, which typically comprises a multi-layer perceptron or Transformer adapter. Its mathematical objective is to learn a mapping function that brings the brain signal feature distribution into alignment with the output distribution of pre-trained text encoders (e.g., CLIP text encoder). This process essentially performs cross-modal feature distillation, forcing heterogeneous brain signals to acquire geometric structures consistent with text in the latent space. Pseudo-text embeddings and soft prompts constitute the core concepts of this paradigm: the pseudo-text embeddings output by the regressor are, in mathematical form, continuous vector sequences with the same dimensionality as real text embeddings, but in physical significance, do not correspond to any specific discrete words, and are commonly viewed as soft prompts. This continuous representation avoids semantic collapse induced by discretization, and can carry subtle intentions or ambiguous perceptions in brain signals that are difficult to describe linguistically. In the latent space conditional injection phase, the pseudo-text embeddings are directly fed into the diffusion model’s denoising network. Since these pseudo-embeddings are already highly aligned with real text embeddings in vector space, the cross-attention layers treat them as standard text conditions and interact with latent visual features (whether convolutional maps or flattened token sequences) across multiple levels, thereby directly guiding the denoising process to generate images conforming to the brain’s intent without requiring explicit linguistic intermediaries.

Representative works of this paradigm include DreamDiffusion [108], BrainCLIP [109] and GWIT [110], which establish an end-to-end generation pathway centered on embedding alignment. DreamDiffusion proposes a two-stage learning framework addressing the low signal-to-noise ratio characteristic of EEG signals: it first pre-trains a neural encoder on large-scale EEG data using masked signal modeling to capture spatiotemporal dependencies in electroencephalographic signals, then, in the fine-tuning stage, trains an adapter to project EEG features into the CLIP text embedding space. By minimizing the distance between EEG embeddings and the corresponding CLIP text embeddings of images, DreamDiffusion successfully generates specific pseudo-text embeddings, enabling the model to achieve robust category-level image reconstruction even with only limited EEG-image paired data, overcoming the limitation that EEG signals are difficult to decode complex sentences. BrainCLIP validates the effectiveness of this paradigm in the fMRI domain, leveraging contrastive learning to project fMRI voxel features into the joint CLIP text-image embedding space. Its core contribution lies in demonstrating that through simple linear or nonlinear projection, brain signals can directly serve as a visual-language bridge, exhibiting superior cross-modal generalization ability in zero-shot classification and generation tasks.

In summary, the cross-modal brain-text latent embedding mapping and injection mechanism successfully circumvents the intermediate step of natural language decoding by constructing a neural-semantic isomorphic mapping. This design not only eliminates the constraints of discrete symbolic systems, but also substantially enhances model inference efficiency and semantic alignment accuracy under low information density signals (e.g., EEG), representing the technological frontier of brain–computer interface generative models toward end-to-end, real-time development.

#### 2.4.4. Analysis of Semantic Reconstruction Image Generation for Brain–Computer Interface Signal Mapping and Injection

**Figure 14:**
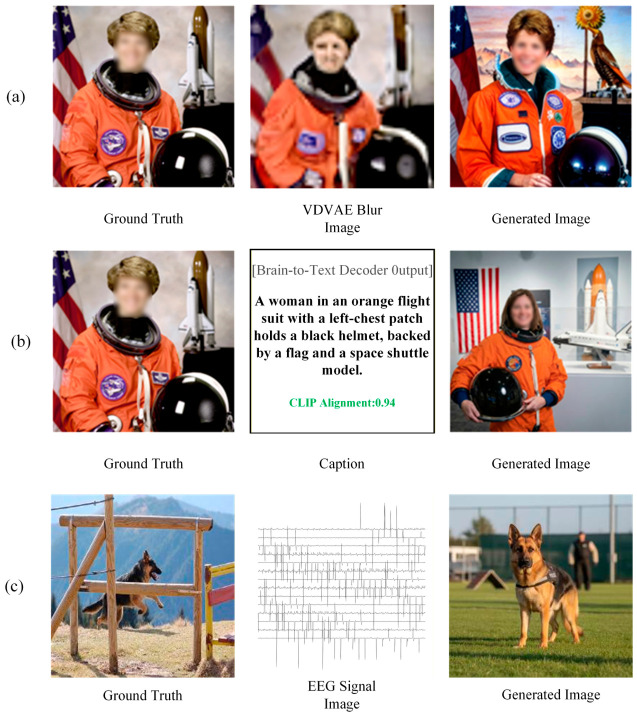
Qualitative comparison of semantic reconstruction via image generation across diverse BCI signal mapping and injection mechanisms: (**a**) Paradigm I (Brain-Diffuser) for pixel-level visual replication; (**b**) Paradigm II (MindSemantix) for semantic-priority reconstruction with core concept alignment; (**c**) Paradigm III (DreamDiffusion) for category-level semantic capture from EEG signals.

As illustrated in Figure 14, the three neural decoding paradigms exhibit differentiated representation tendencies in reconstruction accuracy and semantic capture. Paradigm I (Brain-Diffuser) prioritizes pixel-level visual replication; as shown in Figure 14a, it leverages the structural blueprint of the intermediate VDVAE layer to achieve precise spatial-topological alignment between generated images and physical ground truth. Paradigm II (MindSemantix) manifests as semantic priority with spatial relaxation; as shown in the decoding cards in Figure 14b, while ensuring high-score alignment of core concepts, it exhibits apparent layout drift due to a lack of spatial constraints. Paradigm III (DreamDiffusion) focuses on intention-level semantic mapping; as shown in Figure 14c, it eschews physical restoration in low signal-to-noise ratio environments, achieving precise category-level semantic capture through constructing nonlinear alignment of the neural-text manifold.

In summary, neural mapping paradigms are undergoing technological evolution from “physical feature reconstruction” to “abstract intent alignment”: Paradigm I establishes the fidelity benchmark for visual reconstruction, Paradigm II enhances the analytical depth of cross-modal conceptualization, while Paradigm III provides a robust solution for intent decoding under low information density signals.

### 2.5. Taxonomy and Comprehensive Comparison of Cross-Modal Mapping Methods

To consolidate the diverse methodologies detailed in Section 2.1, Section 2.2, Section 2.3 and Section 2.4, this section synthesizes representative cross-modal generation frameworks. Table 1 categorizes these approaches across seven dimensions, accommodating modal constraints ranging from acoustic rhythms to Brain–Computer Interface (BCI) signals. To ensure objective evaluation and eliminate qualitative ambiguity, computational overhead is strictly quantified via architectural metrics: Control Parameters, Parameter Increment Ratio (Δθ) relative to the base model, and Inference VRAM usage. Analyzing this taxonomy reveals a historical dependence on convolutional bypass structures. Early multi-source spatial conditioning predominantly utilized U-Net backbones (e.g., MIControlNet), where integrating multiple constraints required the physical duplication of adapter networks. This strategy inevitably triggers substantial parameter inflation (e.g., an 84.0% increase) and memory exhaustion, exposing the physical capacity ceilings of spatial addition.

Despite these hardware limitations, the quantitative trajectory in Table 1 indicates that foundational cross-modal mechanisms—such as explicit topological mapping and decoupled attention—remain architecture-independent. These paradigms have been successfully adapted to modern Diffusion Transformers. For instance, by leveraging a unified native token sequence, OminiControl achieves multi-source alignment with a marginal parameter increment of 0.12% atop a 12B-parameter foundation. Similarly, training-free algorithms (e.g., FreeControl) modulate gradient probabilities directly, requiring no additional trainable weights (Δθ=0.0%) and functioning across arbitrary decoders. While migrating to tokenized architectures resolves the structural bloat of U-Nets, transitioning toward heterogeneous collaboration introduces new algorithmic bottlenecks. As physical capacity limits are alleviated by DiT representations, they are immediately replaced by latent mathematical conflicts. Understanding the failure modes of naive multi-modal concatenation in complex collaborative scenarios requires a systematic dissection of these underlying vulnerabilities—specifically, the shift from U-Net capacity saturation to DiT attention competition—which forms the precise focus of Section 2.6.

### 2.6. From Theoretical Foundations to Technical Evolution: The Paradigm Shift Toward Heterogeneous Collaboration

The theoretical frameworks and technical paradigms explored in the preceding sections have thoroughly mapped the mechanics of single-variable control. However, real-world generative systems rarely operate under isolated constraints. To comprehend the current trajectory of controllable generation, it is imperative to analyze both the paradigm shift toward multi-source inputs and the profound architectural bottlenecks exposed during this transition.

#### 2.6.1. The Transition from Single-Variable to Multi-Dimensional Control

As user demands scale toward precise, multi-dimensional scene authoring, a paradigm shift from single-source mapping to multi-source heterogeneous collaboration becomes inevitable. In practical applications, a generation task typically encompasses unstructured text prompts simultaneously with precise spatial geometry (e.g., depth maps) and semantic parameters (e.g., camera poses).

From a mathematical perspective of diffusion models, single-variable control is essentially solving for the conditional score function $\nabla_{x_{t}}\log p(x_{t}|c)$. However, when transitioning to multi-dimensional control with *M* heterogeneous conditions $\left\{c_{1},c_{2},\ldots ,c_{M}\right\}$, the naive approach relies on linearly aggregating individual score estimates via extended Classifier-Free Guidance (CFG):

(2) $${\hat{\epsilon }}_{\theta }(x_{t},t,\{c_{i}\})=\epsilon_{\theta }(x_{t},t,\emptyset )+\sum_{i=1}^{M}\lambda_{i}\left(\epsilon_{\theta }(x_{t},t,c_{i})-\epsilon_{\theta }(x_{t},t,\emptyset )\right)$$

This linear superposition operates on a critical, yet fragile, assumption: that the target data sub-manifolds defined by each condition, denoted as $M_{c_{i}}$, intersect orthogonally and perfectly align in the high-dimensional latent space. In reality, projecting multiple heterogeneous conditions triggers the “curse of dimensionality in control space.” The intersection of these manifolds $\cap_{i=1}^{M}M_{c_{i}}$ is frequently empty or highly distorted. Consequently, the linear addition of gradient vectors leads to severe nonlinear interference, transforming the sampling process into an intricate, highly non-convex multi-objective balancing problem where gradients cancel each other out, leading to structural collapse.

#### 2.6.2. Architectural Bottlenecks: U-Net Asymmetry vs. DiT Attention Competition

To understand the root of these multi-source conflicts, it is necessary to systematically dissect the cross-modal injection bottlenecks of Convolutional Networks (U-Net) and Diffusion Transformers (DiT), particularly regarding computational complexity and capacity limits, as systematically compared in Table 2.

U-Net heavily relies on continuous 2D spatial feature maps and asymmetric cross-attention. In this paradigm, integrating multiple modalities requires deploying parallel bypass encoders, causing the computational complexity to scale linearly with the modality count *M*, expressed as $O(M\cdot N_{img}\cdot N_{cond}\cdot d)$. While this spatial addition preserves topological priors, it inevitably leads to “Capacity Saturation.” The limited cross-attention pathways struggle to align conflicting multi-modal gradients, causing dominant spatial conditions (e.g., dense depth maps) to frequently overwrite weaker textual semantics, ultimately resulting in catastrophic fusion failure and rapid VRAM exhaustion.

Departing from this spatial-centric approach, DiT employs a discrete tokenization mechanism that flattens all conditions into a unified 1D context sequence for global joint self-attention. Although this parameter-free native scaling eliminates the need for massive auxiliary networks, its quadratic computational complexity—$O((N_{img}+\sum_{i=1}^{M}N_{cond,i}{)}^{2}\cdot d)$—introduces a lethal mathematical flaw known as “Attention Competition.” Because tokenization creates a severe numerical imbalance (e.g., over 4000 spatial image tokens competing against a mere 77 text tokens), the standard normalization operation $\mathrm{Softmax}(QK^{T}/\sqrt{d})$ is structurally dominated by the overwhelming mass of $N_{img}$. This imbalance severely dilutes the attention scores allocated to sparse conditioning tokens, triggering critical “Condition Neglection.” Resolving these deep-seated optimization bottlenecks and topological conflicts necessitates a higher-level system architecture, leading directly to the hierarchical fusion and dynamic regulation framework detailed in Section 3.

## 3. Controllable Image Generation with Multi-Source Heterogeneous Coordination

Building upon the foundational “mapping-injection” theoretical framework established for individual modalities in Section 2, this chapter shifts the research focus from single-variable control toward the more complex paradigm of multi-source heterogeneous collaborative generation. To address the representational competition and semantic distortion induced by multi-dimensional control flows within the visual manifold, this chapter systematically deconstructs the problem along three core dimensions: the joint representation and manifold alignment of multi-source heterogeneous inputs; the fusion mechanisms and topological interactions of multi-source conditions; and the conflict and resolution strategies in multi-source heterogeneous collaboration. By synthesizing a novel conceptual hierarchical fusion and dynamic regulation framework oriented toward collaborative generation, this chapter aims to elucidate in depth how a system—under the interplay of multi-dimensional heterogeneous constraints—resolves gradient competition and semantic-spatial conflicts, thereby achieving high fidelity, logical self-consistency, and spatiotemporal coherence in the generated content.

### 3.1. Hierarchical Fusion and Dynamic Regulation Framework for Collaborative Generation

**Figure 15:**
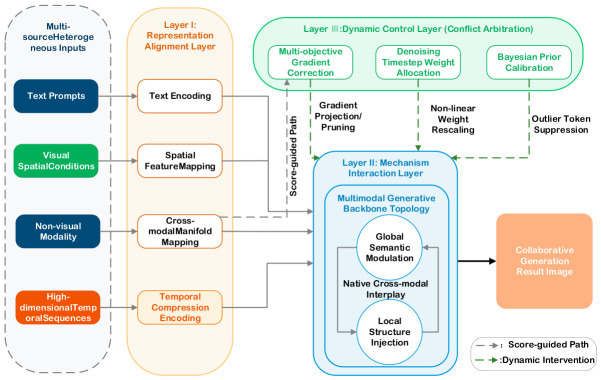
Schematic of the ‘Representation–Interaction–Arbitration’ hierarchical fusion and dynamic regulation framework for multi-source collaborative generation. Functional modules are systematically demarcated via color-mapping to highlight the operational hierarchy: representation alignment (orange, Layer I), mechanism interaction (blue, Layer II), and dynamic regulation (green, Layer III). Directional flow is illustrated by solid black arrows for the primary generative pipeline, with score-guided and dynamic intervention paths indicated by gray and green dashed lines, respectively.

To systematically address the heterogeneity in representation dimension, nonlinearity in interaction dimension, and logical conflicts among control flows for multi-source inputs, this chapter elevates conventional literature summarization by formulating a generalized theoretical hierarchical fusion and dynamic regulation framework. As illustrated in Figure 15, this formulated framework is not a specific algorithmic implementation, but rather an independent conceptual construct aiming to provide a standardized, highly abstract methodology for coordinated generation under heterogeneous conditions. This architecture decomposes the complex fusion process into three logically progressive control layers: the Representation Alignment Layer (Layer I), the Mechanism Interaction Layer (Layer II), and the Dynamic Regulation Layer (Layer III), which collectively constitute the original “Representation–Interaction–Arbitration” hierarchical framework proposed in this review.

(1) Representation Alignment Layer (Layer I): The Collaborative Foundation, aiming to achieve manifold normalization of heterogeneous signals. This layer addresses the inconsistency of input signals in the physical dimension and the semantic level. Its core logic is to normalize raw inputs from different modalities (such as unstructured text, concrete geometric constraints, high-dimensional temporal sequences) into a unified intermediate representation that is operationalizable in the generative space. By executing targeted manifold mapping and temporal compression on signals with different characteristics, it achieves dimensionality reduction and alignment from the raw data space to the latent representation space. This layer establishes the consistency foundation in physical scale and semantic features for subsequent mechanism interaction.
(2) Mechanism Interaction Layer (Layer II): Collaborative Core—defines the interaction topology of multi-source control flows within the generative backbone. Building upon representational normalization, the mechanism interaction layer drives deep fusion between multi-source signals and the denoising backbone network through the design of differentiated interaction paradigms. Based on variations in control density, this layer constructs multiple topological structures, including global semantic modulation, local structural injection, and inter-modal native competition. Its essence lies in defining the routing rules and intervention modalities of information flow within the network, with the aim of fully releasing the control potential of heterogeneous conditions across both the macroscopic dimension of semantic guidance and the microscopic dimension of geometric constraint.
(3) Dynamic Regulation Layer (Layer III): Collaborative Safeguard—responsible for resolving distributional divergence and logical conflicts between control flows. The dynamic regulation layer occupies the outermost position in the framework, fulfilling an “arbitration” function to maintain the logical self-consistency of the generation process. When the gradient directions induced by multi-source constraints in the latent space diverge, or when external instructions come into antagonism with the model’s intrinsic priors, this layer performs dynamic correction through the introduction of systematic intervention mechanisms. Its regulatory means span multiple dimensions—from timestep-level weight allocation and multi-objective gradient correction to adaptive calibration grounded in commonsense distributions. The incorporation of this layer ensures that multi-source collaborative generation maintains a high degree of controlled consistency and perceptual naturalness even under complex constraints.

### 3.2. Joint Representation and Manifold Alignment of Multi-Source Heterogeneous Inputs

**Figure 16:**
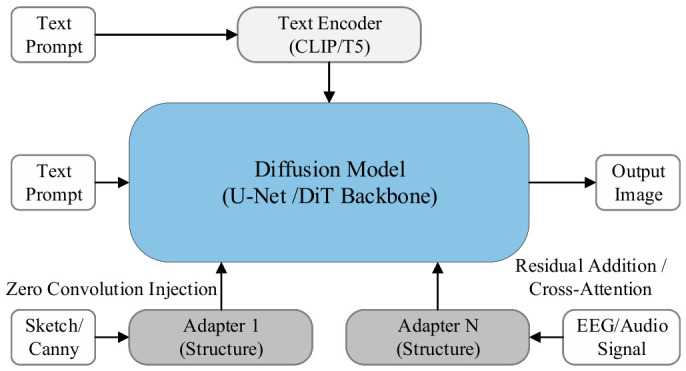
Independent semantic projection via modality adapters.

**Figure 17:**
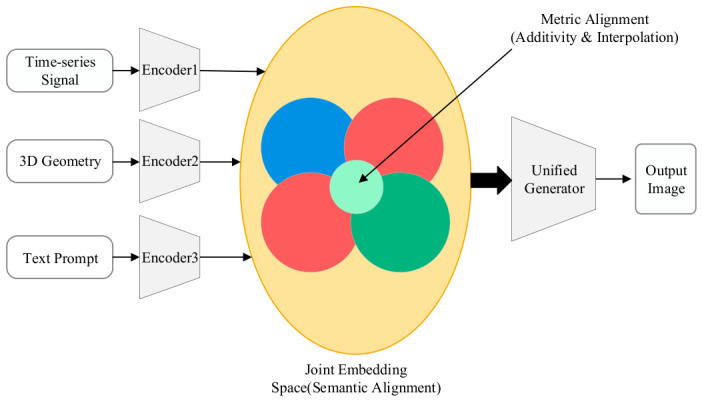
Schematic of the shared manifold representation paradigm via a joint latent space. The color-coded architecture denotes the technical components: the orange background represents the high-dimensional Hilbert space; blue, red, and dark green circles signify the embedding regions of heterogeneous modalities (time-series, 3D geometry, and text, respectively); and the central light green area illustrates the precise metric alignment for additivity and interpolation.

**Figure 18:**
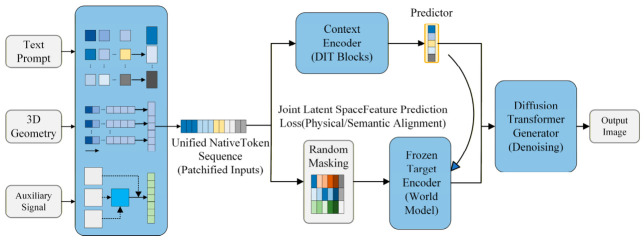
Deep representation paradigm via predictive architecture and native sequences. Heterogeneous multi-source inputs are flattened into a unified token sequence, where the multi-colored token blocks illustrate the diverse feature representations and the random masking process. For the directional flow, solid arrows delineate the primary data pipeline and joint latent space prediction path; internal dashed arrows indicate modality-specific mapping within the auxiliary block; and the curved arrow designates the feature prediction guidance provided by the frozen target encoder (i.e., the world model).

Representational heterogeneity constitutes the foremost obstacle to achieving multi-source collaborative generation. Input data spans two-dimensional visual maps, one-dimensional sequential signals, and high-dimensional topological structures; how to map conditions of varying physical properties and semantic granularity into a unified, operable space forms the foundational basis for determining the consistency of the fusion system. This section systematically elucidates how multi-source heterogeneous inputs are transformed into consistent representations interpretable by the generative model, across two dimensions: the topological evolution of encoder architectures and the metric alignment of latent spaces. In accordance with the progression of technical approaches, this section organizes the discussion into three successive stages:

(1) Modality Representation Paradigm Based on Independent Semantic Projection. As illustrated in Figure 16, this paradigm is dedicated to constructing modality-specific adapters, aiming to unidirectionally project heterogeneous signals into the latent feature space of the generative model. Representative works in this category include T2I-Adapter and similar spatial control flow conversion frameworks, as well as “Guess What I Think” in the domain of physiological signals, which employs specialized adaptation layers to map EEG features into continuous prompt embeddings. The architectural characteristic of this paradigm is the independent introduction of constraints through parallel control branches, while preserving the primary text backbone pipeline (e.g., CLIP/T5). This encompasses structured spatial injection via zero convolution and semantic/temporal feature collaboration via cross-attention. The mechanistic core lies in the use of a decoupled adaptation strategy to ensure non-invasive constraint injection—that is, achieving flexible modulation of control intensity through modular components without perturbing the parameter distribution of the pre-trained backbone model. However, the limitation of this paradigm is that the encoding process of each modality lacks cross-dimensional metric association, causing each control flow to manifest as an “isolated mapping” on the mathematical manifold. This restricts the efficacy of feature interaction in complex multi-source collaborative scenarios.
(2) Shared Manifold Representation Paradigm Based on Joint Latent Space. As illustrated in Figure 17, to overcome the interoperability bottleneck caused by independent mapping, this paradigm is dedicated to constructing a unified cross-modal metric space. Landmark works such as ImageBind [113] and LanguageBind [114] anchor heterogeneous modalities—including sequential signals, 3D geometry, and text—within a high-dimensional Hilbert space through large-scale contrastive learning, achieving deep semantic alignment. The theoretical essence of this paradigm lies in the construction of cross-modal metric equivalence: by realizing metric alignment within a shared manifold, feature vectors are endowed with mathematical additivity and interpolability. This standardized representation not only eliminates the incommensurability of physical dimensions, but also provides a mechanistic guarantee for dynamically weighted collaboration among multi-source signals within the same semantic dimension, while directly adapting to unified generators with native token interaction capabilities—such as DiT [3] and Lumina-T2X [115]. Despite significantly enhancing collaborative efficiency at the semantic level, this pathway still faces challenges in handling fine-grained feature alignment and the physically consistent expression of high-noise signals such as physiological signals.
(3) Deep Representation Paradigm Based on Predictive Architecture and Native Sequences. To address the limitations of preceding approaches in logical consistency and noise robustness, cutting-edge research represented by TI-JEPA [116] and OminiControl [73] is leading a technical evolution toward predictive alignment. As illustrated in Figure 18, this paradigm abandons the cumbersome external independent encoder architecture in favor of native tokenization and flattening, converting heterogeneous inputs—including text prompts, 3D geometry, and auxiliary signals—into a unified native token sequence. The architectural essence lies in the introduction of a latent space feature prediction mechanism: leveraging a context encoder in conjunction with a random masking strategy, a predictor computes the joint latent space feature prediction loss under the guidance of a frozen target encoder (i.e., the world model). The methodological core of this paradigm is to enhance the system’s fault tolerance against heterogeneous conflicting signals through predictive representation, while eliminating the information entropy increase caused by external mapping by leveraging the native attention mechanism internal to the generative model—thereby achieving an architectural leap from “surface-level semantic equivalence” to “deep physical logic consistency.”

### 3.3. Fusion Mechanisms and Topological Interactions of Multi-Source Conditions

**Figure 19:**
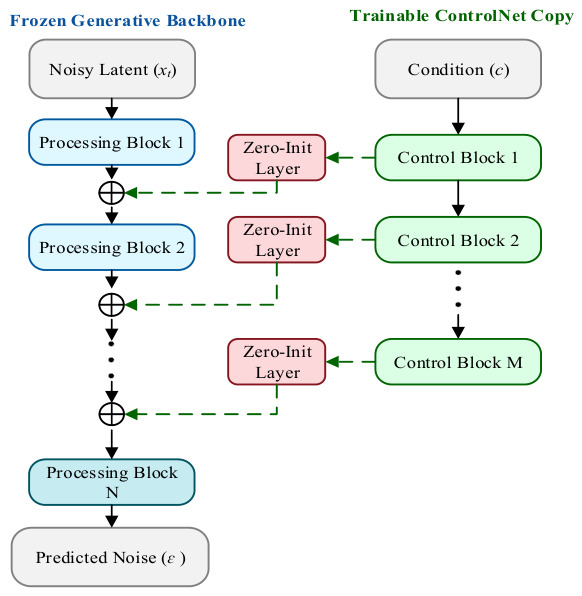
Spatial bias injection architecture based on residual superposition (ControlNet as the canonical paradigm). Solid black arrows delineate the primary data flow within both the frozen generative backbone and the trainable ControlNet copy. Green dashed arrows illustrate the residual feature injection paths, mapping control signals through zero-initialized projection layers into the main network.

This section aims to conduct a systematic mechanistic analysis and empirical examination of existing multi-source collaborative paradigms from the perspective of the specific operator implementations underlying fusion mechanisms and the pathway construction of topological interactions:

(1) Additive Embedding Paradigm Based on Spatial Bias. This paradigm primarily serves strong structural constraints, with its core logic residing in the injection of heterogeneous features as bias terms into the denoising backbone via residual connections. Its mechanistic essence lies in achieving linear feature superposition in the feature space, with representative works spanning from the full-parameter branch of ControlNet (as shown in Figure 19) to the lightweight interface of T2I-Adapter. Such methods leverage zero-initialized projection operators to achieve rigid fusion of spatial constraints at the corresponding processing blocks of the generative backbone (e.g., U-Net encoders or DiT hidden layers). While this mechanism ensures exceptionally high structural control precision, in multi-source collaborative scenarios, additive injection can readily induce numerical overflow in the feature stream within deeper network layers, thereby triggering the control flow conflicts discussed in Section 3.4.
(2) Flexible Intervention Fusion Paradigm Based on Attention Modulation. To overcome the limitations of additive injection when handling unstructured information, fusion mechanisms have progressively shifted toward “flexible intervention” approaches grounded in attention weight modulation or gradient guidance. This paradigm is represented by IP-Adapter [67] and Uni-ControlNet [117], where heterogeneous features serve as the Key and Value matrices of cross-attention (with the main backbone latent acting as the Query), achieving multiplicative intervention on the semantic distribution of backbone tokens. The methodological core lies in leveraging this asymmetric attention routing to realize feature modulation of multi-source signals. Furthermore, as shown in Algorithm 1, this domain has given rise to training-free score guidance algorithms exemplified by FreeControl [112], which achieves non-parametric correction of the generation trajectory by introducing gradient operators on the inference side. While this paradigm enhances the flexibility of semantic collaboration, its inherently “dominant-subordinate interaction” nature—structurally determined by the bipartite routing mechanism of cross-attention—continues to constrain the modeling depth of long-range dependencies between heterogeneous modalities.
**Algorithm 1**: Score-Guided Late FusionInput:Conditions:*c*_1_,…, *c_n_*; Pre-trained model:model; Total diffusion timesteps:T; Weight function *w_i_(t)*Output:Generated image *x*_0_1:$x_{T}\leftarrow sample\_gaussian\_noise()$2: for t in reversed(range(T)): 3:$\epsilon_{1}=\mathrm{model}(x_{t},c_{1},t)$4:$\epsilon_{2}=\mathrm{model}(x_{t},c_{2},t)$5:…6:$\epsilon_{n}=\mathrm{model}(x_{t},c_{n},t)$7:$\epsilon_{1}=\mathrm{normalize}(\epsilon_{1})$8:$\epsilon_{2}=\mathrm{normalize}(\epsilon_{2})$9:…10:$\epsilon_{n}=\mathrm{normalize}(\epsilon_{n})$11:$\epsilon_{\mathrm{combined}}=w_{1}(t)\cdot \epsilon_{1}+w_{2}(t)\cdot \epsilon_{2}+\ldots +w_{n}(t)\cdot \epsilon_{n}$12:$x_{t-1}=\mathrm{denoise}\_\mathrm{step}(x_{t},\epsilon_{\mathrm{combined}},t)$13:end for14:return x_0_
(3) Native Topological Fusion Paradigm Based on Full Attention. To address the bottlenecks of preceding approaches in injection depth and interaction dimensionality, cutting-edge architectures represented by FLUX.2 [118] and OminiControl [73] have achieved a paradigm leap from “side-branch injection” to “native interaction.” As illustrated in Figure 18, this paradigm entirely abandons external independent modules, representing all heterogeneous conditions as peer token sequences and concatenating them into a single unified context stream before feeding them into a multimodal diffusion Transformer (MM-DiT). Its theoretical essence lies in the construction of a “feature competition” mechanism driven entirely by Self-Attention. Explicitly distinguishing itself from the asymmetric routing of cross-attention (where conditions are passively queried as Key/Value pairs), this architecture leverages the omnidirectional self-attention operator internal to the backbone to guide patches of different modalities in engaging in token-level nonlinear competition within a unified manifold space. This highly integrated topological structure not only eliminates the feature mismatch issues inherent in traditional architectures, but also provides the most direct physical substrate for resolving the dynamic weight optimization and conflict arbitration discussed in Section 3.4.

### 3.4. Conflict and Resolution Strategies in Multi-Source Heterogeneous Collaboration

#### 3.4.1. Mathematical Formalization and Theoretical Verification of Gradient Conflicts

A rigorous mathematical formulation of gradient competition is essential to decode the fusion instability inherent in multi-source scenarios. Within the score-matching framework, injecting heterogeneous control signals (e.g., $c_{i}$ and $c_{j}$) generates competing driving gradients in the latent vector space, formally defined as $G_{i}=\nabla_{z}\log p(z|c_{i})$. This competition occurs when these vector fields direct the denoising trajectory toward divergent sub-manifolds. Drawing inspiration from MIControlNet [111] while addressing theoretical controversies surrounding the symmetry of the score function Jacobian matrix, we formalize this directional divergence into a computable proxy: the Asymmetry Index (A). It explicitly maps the vector-space gradient conflict between any pair of heterogeneous signals ($G_{i}$ and $G_{j}$) into a normalized scalar metric based on cosine distance:

(3) $$A(G_{i},G_{j})=1-\frac{\langle G_{i},G_{j}\rangle }{\left||G_{i}||\cdot ||G_{j}|\right|}$$

Mathematically, when the semantic and spatial gradients are orthogonal (cosθ=0),A=1. However, when active semantic-space antagonism manifests (i.e., cosθ<0, gradient angle > 90°), the index strictly exceeds 1 (A>1). This generalized metric seamlessly bridges theoretical cosine similarity with empirical instability, where an escalating Asymmetry Index invariably triggers catastrophic manifold disruption.

**Figure 20:**
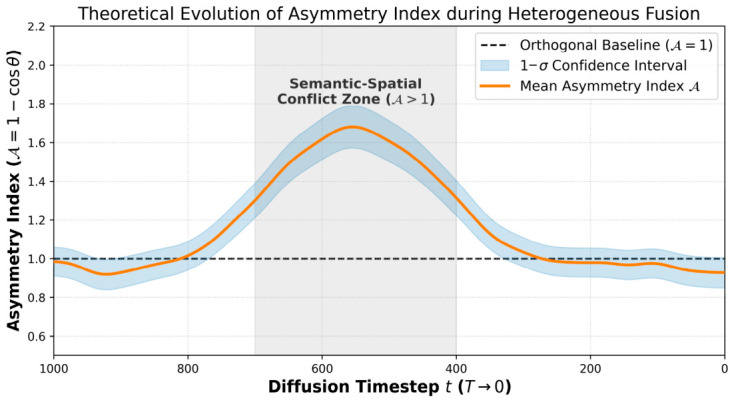
Theoretical experiment on gradient competition verifying the semantic-spatial conflict hypothesis. The orange curve represents the mean trajectory of the Asymmetry Index, while the light blue shaded area denotes theconfidence interval. The gray shaded region indicates the Semantic-Spatial Conflict Zone, where the index significantly exceeds the orthogonal baseline (dashed line) during the critical mid-stage of the diffusion process.

To computationally verify this generalized conflict hypothesis, we designed a small theoretical experiment tracking the statistical distribution of the gradient angle across the reverse diffusion process. To isolate the fundamental theoretical dynamics from specific architectural noise, we simulated the latent score trajectories using a Monte Carlo synthetic distribution model (Figure 20). We instantiate this simulation using a canonical semantic-spatial collision as a generalized case study (where $G_{i}=g_{semantic}$ and $G_{j}=g_{spatial}$). While this pure mathematical simulation models the invisible vector-space collisions, the macroscopic visual consequences of such typical antagonisms (e.g., structural collapse, spatial dislocation) will be broadly observed and classified later in the empirical taxonomy of Section 3.4.2. The trajectory of the mean Asymmetry Index and its 1 − σ confidence variance reveals a distinct multiphase evolution. During the initial global noise reduction (as *t* descends through [800,1000]), the semantic and spatial gradients remain largely orthogonal (cosθ≈0, yielding A≈1). As the generation advances into the critical layout-formation stage (where *t* falls within [400,700]), a severe Semantic-Spatial Conflict Zone emerges. Here, the gradient angle diverges into a deep negative correlation (cosθ<−0.6), forcing the Asymmetry Index to spike significantly above 1.6 (A>1.6). This statistical trajectory rigorously substantiates that such implicit mathematical antagonisms—specifically the sustained spike in asymmetry during mid-stage denoising—act as the fundamental drivers of explicit fusion instability.

#### 3.4.2. Taxonomy of Conflicts in Multi-Source Heterogeneous Collaboration

**Figure 21:**
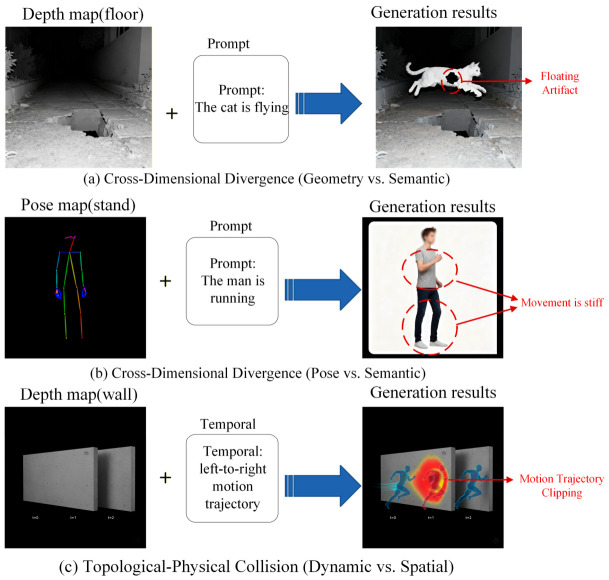
Visual analysis of representative explicit spatial conflicts via cross-dimensional and topological manifold disruption. (**a**) Cross-Dimensional Divergence (Geometry vs. Semantic): Solid red circles identify “floating artifacts” caused by semantic-spatial mismatch. (**b**) Cross-Dimensional Divergence (Pose vs. Semantic): Red dashed circles diagnose “stiff movement” resulting from gradient antagonism. (**c**) Topological-Physical Collision (Dynamic vs. Spatial): Red arrows and the color-coded collision zone indicate “motion trajectory clipping” at rigid spatial boundaries. Across all panels, red annotations serve as visual diagnostic markers of fusion instability.

The conflicts in multi-source collaborative generation are fundamentally probabilistic distribution antagonisms induced by heterogeneous control flows within the denoising manifold. Transcending the surface-level phenomena of modality combinations reviewed in previous sections, this section introduces a novel conflict taxonomy proposed in this review. To clearly distinguish our original theoretical contributions from existing literature summaries, our proposed taxonomy categorizes latent antagonisms into two primary categories—Explicit Spatial Conflicts and Implicit Logical Conflicts—comprising the following four abstract dimensions based on information interaction logic and gradient dynamics. While Figure 21 provides a visual comparison of the more prominent explicit spatial conflicts (Dimensions 2 and 3) by presenting concrete case studies of fusion instability, the implicit logical conflicts (Dimensions 1 and 4) primarily manifest as latent-space antagonisms that are less conducive to direct visual representation.

(1) Categorized as an implicit logical conflict, Intra-Modal Semantic Dissonance reflects logical mutual exclusion within homogeneous semantic space. This dimension focuses on representational conflicts internal to unstructured semantic flows. The mechanistic essence lies in the non-intersecting probability that distributions induced by heterogeneous semantic instructions in the latent space, causing the joint distribution to fail to converge toward a logically self-consistent centralized cluster. As a conceptual case study of semantic competition, when the system receives textually mutually exclusive physical attribute instructions—such as “extreme cold climate” and “tropical vegetation”—the generator manifests as manifold oscillation along the sampling trajectory, ultimately triggering semantic drift or logical collapse of the generated content.
(2) As a primary explicit spatial conflict, Cross-Dimensional Alignment Divergence represents representational mismatch across dimensional manifolds. This dimension is defined as the distributional mismatch between “higher-order semantic attributes” and “lower-order spatial priors,” and constitutes the core driver of guidance conflicts. To demonstrate how this manifests in real multimodal generation systems, consider the concrete case studies of fusion instability in Figure 21a,b. When the content attributes defined by semantic tokens and the spatial distributions anchored by geometric maps produce non-orthogonal projections, a response vacuum emerges. In Figure 21a, a severe gradient conflict occurs when the semantic prompt (“The cat is flying”) contradicts the grounded geometry of the provided depth map. The network fails to reconcile the mid-air semantic trajectory with the physical floor, empirically resulting in a spatial dislocation diagnosed as a “floating artifact” without coherent shadows. Likewise, Figure 21b illustrates a classic scenario of semantic competition: A dynamic semantic intent (“The man is running”) is forcibly overwritten by the excessive constraint of a static standing pose prior. Mathematically, this manifests as an extreme angular divergence between the semantic guidance gradient and the spatial control gradient acting on the diffusion backbone (gradient angle > 90°), producing a paradoxical biomechanical distortion where the generated entity exhibits “stiff movement” (a standing-run posture).
(3) Furthermore, as another explicit spatial conflict, Topological–Physical Constraint Collision addresses anchor point conflicts in topological physical structures. This dimension encompasses the topological overlap and mutual exclusion of strong geometric constraint sources at the same physical site. In multi-source collaborative scenarios, spatial anchors extracted by heterogeneous sensors or visual operators may generate mutually conflicting guidance at the voxel level. A concrete case study of this topological fusion instability is visually diagnosed in Figure 21c. When a temporal condition specifies a continuous “left-to-right motion trajectory,” it collides directly with a static 3D depth map representing rigid, occluding walls. The continuous temporal gradient attempts to propagate features across frames but is abruptly truncated by the hard geometric boundaries of the spatial depth gradient. These manifest as “motion trajectory clipping” or loss of spatiotemporal continuity, revealing the robustness bottleneck of heterogeneous signals in handling high-dimensional physical logic consistency.
(4) Concluding the conceptual taxonomy proposed in this review as a final implicit logical conflict, extrinsic–intrinsic distribution antagonism denotes distributional contention between external constraints and intrinsic priors. Serving as the closed-loop dimension of the taxonomy, this conflict originates from the contention between user-defined external constraints and the inherent “world model” priors of the generative model. Even when the input conditions are mutually logically consistent, once their combined distribution deviates from the commonsense distribution learned by the pre-trained model on large-scale datasets—such as the concrete scenarios of unnatural lighting or extreme anatomical configurations—the model’s strong internal priors generate a rejection response against the control flows. How to simultaneously ensure controlled consistency while preserving the distributional naturalness of generated content has become the central research focus of current conflict resolution algorithms.

#### 3.4.3. Resolution of Multi-Source Collaborative Conflicts

**Figure 22:**
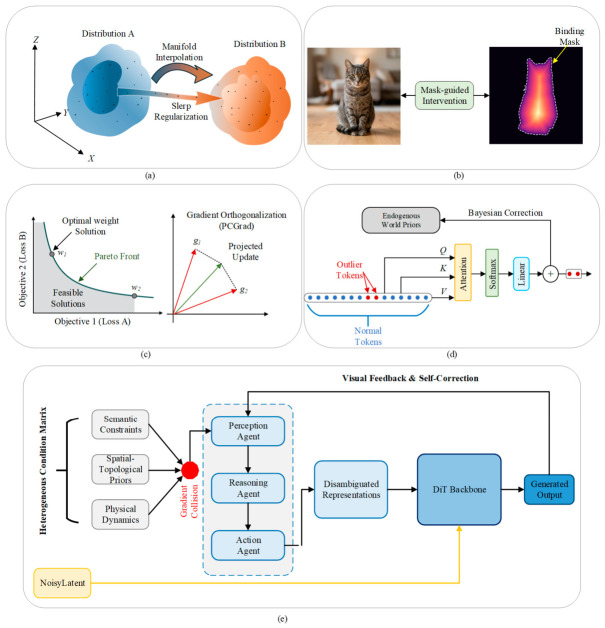
Schematic of hierarchical resolution strategies for multi-source collaborative conflicts. (**a**) Regularized smoothing and reprojection via manifold interpolation and Slerp regularization; (**b**) spatial decoupling and localized attention modulation using mask-guided intervention and weight rescaling; (**c**) multi-objective optimization through gradient orthogonalization (PCGrad) and Pareto front search; (**d**) Bayesian prior calibration utilizing attention bias tuning and world-prior-guided adaptive correction; (**e**) cognitive routing via multi-modal agents featuring perception-reasoning-action loops and visual feedback self-correction.

The central challenge of multi-source collaborative generation lies in the fact that heterogeneous control flows frequently induce latent representation conflicts within the denoising manifold. This phenomenon is fundamentally attributable to manifold distributional divergence arising from the conflicting probability distributions induced by different constraint conditions. In response to the aforementioned manifold inconsistency problem:

(1) Regularized Smoothing and Reprojection of Homogeneous Semantic Manifolds. To mitigate the inherent intra-modal semantic dissonance, this approach addresses logical mutual exclusion within the semantic space; the resolution focus lies in eliminating severe oscillations along the sampling trajectory through latent distribution regularization. As illustrated in Figure 22a, the core intervention approaches include: ① Manifold Interpolation in Vector Space—for instance, composable diffusion [119] proposes leveraging compositional logic based on energy-based models (EBMs), applying spherical linear interpolation (Slerp) operators during the sampling stage to smooth mutually exclusive semantic features and forcibly guide the sampling path back toward a consistent latent distribution region; ② Contrastive Semantic Guidance Constraints—exemplified by semantic guidance [120] (SeG), which explicitly constrains the model to avoid logical paradox regions at inference time by introducing a negative semantic penalty term. This strategy rigorously suppresses semantic drift on the input side, ensuring generative logical robustness under complex semantic combinations.
(2) Spatial Decoupling and Localized Attention Modulation for Cross-Dimensional Interaction. To bridge the cross-dimensional alignment divergence between semantic attributes and geometric priors, this mechanism focuses on the precise decoupling and reshaping of cross-attention maps. As illustrated in Figure 22b, the principal technical pathways include: ① Mask-Guided Local Intervention—exemplified by MultiDiffusion [121] and GLIGEN [122], which apply explicit pruning to the activation distributions of semantic tokens using spatial priors or binding masks, suppressing the redundant diffusion of attribute features into non-target regions; ② Semantic Response Enhancement and Weight Rescaling—targeting the “response vacuum” phenomenon in attention maps (i.e., the failure of critical targets to be activated), Attend-and-Excite [123] introduces a nonlinear weight rescaling mechanism that iteratively optimizes and enhances the semantic saliency of target regions. This pathway mitigates the risk of object vanishment from the dimension of spatial topology, achieving high-dimensional alignment between semantic content and geometric positioning.
(3) Gradient Orthogonalization and Pareto Optimization for Topological Anchor Point Conflicts (Gradient Orthogonalization & Pareto Optimization) [124]. To resolve topological–physical constraint collisions induced by overlapping spatial anchors, this resolution strategy introduces conflicting gradient reprojection techniques drawn from multi-objective optimization theory. As illustrated in Figure 22c, representative algorithms such as FreeControl [112] execute the following interventions: ① Gradient Flow Orthogonalization Projection—drawing on the PCGrad paradigm from multi-task learning [125], when the induced gradient vectors of multi-source control flows form an angular divergence greater than 90°, the orthogonal components between gradients are computed and conflicting directions are eliminated, ensuring logical consistency in the update direction; ② Pareto Front Search Based on Pareto Optimality—dynamically solving for the optimal weight set satisfying multiple constraints within the inference loop, preventing dominant constraints from masking weak control signals [126]. This intervention scheme, grounded in system dynamics, preserves control precision under complex topological architectures.
(4) Bayesian Prior Calibration and Adaptive Correction Guided by Intrinsic Distribution [127]. To reconcile the extrinsic–intrinsic distribution antagonism, the cutting-edge intervention pathway is advancing toward prior-preserving sampling [128]. As illustrated in Figure 22d, building upon the native topological architectures of multimodal diffusion Transformers (MM-DiT)—such as FLUX.1 and OminiControl [73]—the resolution mechanism achieves intrinsic weight arbitration through: ① Attention Bias Adaptive Tuning—leveraging the physical world priors acquired by the pre-trained backbone during large-scale pre-training to automatically identify and suppress outlier tokens that violate the laws of natural distribution. For instance, OminiControl dynamically corrects external control flows at inference time through a decoupled attention mechanism, preventing them from compromising the model’s inherent image fidelity [129]. ② Dynamic Scale Adjustment of Classifier-Free Guidance (CFG)—drawing on the concept of Perturbed Attention Guidance (PAG), which dynamically scales down the external guidance intensity, generating logical conflicts in real time, based on the degree of deviation of the generated content from the Bayesian prior distribution [130]. This mechanism resolves the fundamental opposition between controlled consistency and generative naturalness at the level of the underlying architecture, representing the current highest technical form of multi-source collaborative conflict resolution.
(5) Cognitive Routing and Closed-Loop Planning via Multi-Modal Agents (MM-Agents). To overcome the acute gradient collapse caused by bottom-up interventions on extremely conflicting inputs—where semantic constraints (e.g., unstructured text prompts) logically contradict spatial-topological priors (e.g., rigidly anchored depth maps) or physical dynamics—the cutting-edge paradigm transitions toward top-down cognitive planning. As illustrated in Figure 22e, mimicking a “System 2” reasoning process, Multi-Modal Agents act as intelligent “front-end routers” that parse these spatial-semantic conflicts and explicitly reason about physical commonsense to generate disambiguated intermediate representations before diffusion denoising begins. This cognitive trajectory is pioneered by milestones like MCCD [131], which decouples overlapping entities into precise layout priors, and is further advanced by recent closed-loop frameworks such as AgentComp [132] and Agentic Retoucher [133]. These state-of-the-art architectures deploy a tripartite system—comprising Perception, Reasoning, and Action Agents—to iteratively localize distortions, perform human-aligned diagnostic inference, and execute rendering via DiT backbones. By utilizing autonomous agents to pre-assign non-overlapping spatial constraints and self-correct through visual feedback, this paradigm structurally bypasses the severe “attention competition” inherent in raw DiT sequence concatenation, representing the current zenith of multi-source heterogeneous prompt resolution.

#### 3.4.4. Schematic of Hierarchical Resolution Strategies for Multi-Source Collaborative Conflicts

In summary, the conflict resolution mechanisms underlying multi-source heterogeneous collaborative generation are undergoing a profound evolution: moving from bottom-up mathematical interventions—such as gradient orthogonalization and Bayesian prior calibration—toward a top-down, cognitive regulation paradigm driven by Multi-Modal Agents. By elevating the resolution process from raw latent space arithmetic to explicit “System 2” logical reasoning and closed-loop visual feedback, these state-of-the-art systems have become capable of dynamically mitigating manifold divergence and decoupling acute spatial-semantic conflicts. Nevertheless, despite these architectural leaps, a complex trade-off relationship inherently persists between strict control fidelity and generative naturalness. This methodological maturity urgently calls for a scientific evaluation framework to quantify the practical efficacy of such “conflict resolution” strategies. Accordingly, Section 4 will conduct an in-depth performance assessment and applied analysis of multi-source collaborative systems across three dimensions: cross-modal dedicated datasets, multi-dimensional consistency metrics, and technical selection strategies.

## 4. Performance Analysis of Controllable Image Generation via Cross-Modal Mapping Injection and Multi-Source Heterogeneous Collaboration

### 4.1. Heterogeneous Collaboration Benchmarks and Metric Systems for Mapping and Injection Paradigms

The conflicts in multi-source collaborative generation are fundamentally rooted in probability distribution inconsistencies and gradient interference induced by heterogeneous control flows within the denoising manifold. To quantitatively analyze these dynamics, this section organizes and synthesizes existing evaluation criteria across two dimensions—dataset clustering and metric standardization—thereby distilling an orthogonal evaluation paradigm that encompasses the full spectrum of modality scenarios and lays the empirical foundation for a unified normalization protocol introduced in Section 4.2.

#### 4.1.1. Heterogeneous Benchmark Datasets for Mapping Injection and Collaborative Consistency

Based on the physical properties and semantic density of different constraint modalities, this review partitions mainstream benchmarks into five core collaborative clusters:

(1) Visual-Centric Cluster: Focused on validating the model’s capacity to maintain spatial control precision. Represented by MultiGen-20M [134] and COCO-Stuff [135], this cluster is primarily used to evaluate the spatial layout fidelity of diffusion models when processing high-density visual priors.
(2) Spatio-Temporal Acoustic Cluster: Focused on evaluating audio-visual synchronization and cross-modal semantic mapping. Datasets such as VGGSound [136] and AudioCaps [137] are used to validate the spatiotemporal alignment performance of collaborative systems when handling highly dynamic acoustic signals.
(3) Spatial-Geometric Cluster: Focused on evaluating three-dimensional topological consistency and manifold reconstruction robustness. Objaverse [138] and GSO [139] are employed to validate the structural fidelity of 3D generation paradigms under geometric constraints.
(4) Neural-Semantic Cluster: Focused on nonlinear mapping from biological signals to the visual manifold. NSD (Natural Scenes Dataset) [140] and EEG-Image [141] are used to validate the manifold alignment of neural decoding models under extremely low signal-to-noise ratio constraints.
(5) Full-Modal Interwoven Cluster: Focused on validating multi-source instruction following and logical arbitration capabilities. Represented by the CoDi-2 Set [142], this cluster evaluates the collaborative consistency of general-purpose paradigms when handling complex and conflicting interwoven instructions.

#### 4.1.2. Multi-Dimensional Evaluation Metrics for Mapping Fidelity and Collaborative Injection Efficacy

**Figure 23:**
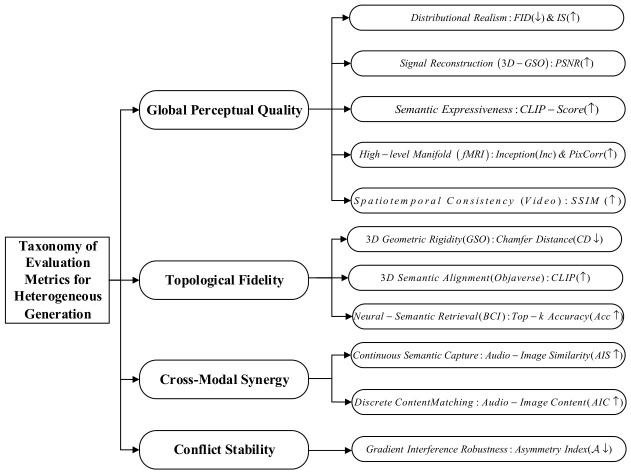
Taxonomy of Evaluation Metrics for Heterogeneous Cross-Modal Image Generation. The symbols (↑) and (↓) denote whether a higher or lower metric value indicates better performance, respectively.

To quantitatively resolve the gradient interference and distribution inconsistencies within multi-source heterogeneous generation, relying on isolated, unorganized metrics is fundamentally insufficient. To directly address the incommensurability across diverse modality domains and provide clear metric definitions, this review establishes a structured evaluation taxonomy, as systematically illustrated in Figure 23.

Rather than a flat enumeration, our taxonomy strictly bifurcates the evaluation criteria into four orthogonal macroscopic dimensions. It is crucial to emphasize that these four metric dimensions do not hold a simplistic one-to-one mapping with the five dataset clusters identified in Section 4.1.1. Instead, they form a dense, orthogonal evaluation matrix. For instance, the Spatial-Geometric cluster is quantitatively resolved through the intersection of the Quality and Fidelity dimensions, whereas the Full-Modal Interwoven cluster necessitates the explicit activation of the Stability dimension to diagnose complex gradient conflicts. Each dimension is explicitly designed to capture a specific physical generative bottleneck across these benchmarks:

(1) Global Perceptual Quality: Evaluates the overarching distributional realism (via FID and IS), structural consistency (SSIM for video), and signal reconstruction (PSNR for geometry). For complex neural-semantic manifolds, it further incorporates Inception (Inc) and PixCorr to assess high-level manifold alignment.
(2) Topological Fidelity (Domain-Specific): Rigorously quantifies the strict preservation of physical and semantic constraints, ranging from geometric structural rigidity in 3D generation (Chamfer Distance) and semantic alignment (CLIP) to complex neural-semantic retrieval in BCI decoding (Top-*k* Accuracy).
(3) Cross-Modal Synergy: Specifically tailored for temporally highly interwoven scenarios (e.g., Audio-driven tasks), deploying AIS and AIC to monitor continuous and discrete semantic synchronization.
(4) Conflict Stability: Uniquely introduces the Asymmetry Index (A), as mathematically formalized in Section 3.4.1, to diagnose architectural vulnerabilities against multi-source gradient tugs-of-war.

Bridging to Unified Benchmarking: While the metric taxonomy outlined in Figure 23 explicitly defines the targeted evaluation proxies for isolated vertical domains, their disparate physical meanings and divergent numerical scales preclude direct cross-domain comparison. Consequently, the establishment of this taxonomy serves as the foundational mathematical prerequisite for Section 4.2, where these proxies are ingested into the Ordinal Mapping Normalization Protocol (OMNP) to compute standardized relative gains (ΔG).

### 4.2. Performance Analysis of Image Generation Models for Cross-Modal Mapping and Collaborative Injection

Transitioning from single-modality mapping to collaborative generation necessitates a rigorous evaluation of the inherent trade-offs between generative quality and controllable precision. A fundamental challenge in benchmarking these cross-modal models lies in the heterogeneity of experimental protocols; metrics compiled directly from original publications (e.g., PSNR versus FID) exhibit substantial magnitude variances and incommensurable physical units, precluding direct absolute comparisons. To resolve this comparative limitation and rigorously quantify the architectural efficacy of distinct mapping paradigms, this section establishes a unified mathematical evaluation framework.

#### 4.2.1. The Ordinal Mapping Normalization Protocol (OMNP) for Heterogeneous Evaluation

To conduct an in-depth analysis of the interaction mechanisms of heterogeneous control flows within the denoising manifold, this section introduces the Ordinal Mapping Normalization Protocol (OMNP) proposed in this review. Distinguishing itself from the review of existing disparate evaluation metrics, this original protocol aims to overcome the incommensurability of heterogeneous evaluation metrics across diverse research domains. Building upon the explicit evaluation taxonomy established in Section 4.1.2, OMNP operationalizes the concept of Functional Equivalence. It orthogonally abstracts those disparate domain-specific metrics into a macroscopic multidimensional coordinate system, utilizing the previously defined axes of Quality, Fidelity (or Synergy), and Stability.

Fundamentally, the dimensionality of the OMNP space dynamically adapts to the architectural complexity. In single-modality injection scenarios, the trade-off fundamentally occurs between the generative prior and a singular condition; thus, the architectural efficacy is strictly resolved within the 2D Pareto plane of Quality and Fidelity. However, when confronting multi-source heterogeneous collaborative scenarios, inter-conditional gradient interferences trigger a topological expansion, activating Stability as the critical third dimension (as analyzed in Section 4.2.3).

To eliminate dataset-induced biases, OMNP strictly extracts the dataset-specific relative gain (ΔG) against established local baseline anchors rather than comparing absolute values. For any raw relative performance gain ΔG extracted from empirical literature benchmarks (computed here as the absolute numerical improvement against the baseline), its normalized score $S_{norm}$ within a unified competitive coordinate system is formulated as. It is worth noting that due to the intra-dataset normalization factor $\log(1+|\Delta G{|}_{max})$ in the denominator, utilizing absolute numerical improvements is mathematically strictly equivalent to utilizing percentage improvements (as linear baseline scaling factors elegantly cancel out), ensuring robust relative benchmarking.

(4) $$S_{norm}=S_{base}+\mathrm{sgn}(\Delta G)\cdot \Psi \left(\frac{\log(1+|\Delta G|)}{\log(1+|\Delta G{|}_{max})}\right)\times \omega$$

Here, $S_{base}$ designates the established performance baseline anchor score. The signum function sgn(ΔG) is strictly employed to mathematically capture positive synergistic gains or antagonistic degradations (resulting in $S_{norm}<0.5$). The logarithmic term acts as a percentile rank mapping function (Ψ), where $|\Delta G{|}_{max}$ denotes the maximum absolute gain boundary observed under the specific task benchmark, ensuring a strictly bounded and smooth score distribution. Finally, ω=0.5 functions as the environmental sensitivity weight, which can be tailored for different topological conflict scenarios. Consequently, this originally proposed protocol rigorously projects discrete, incommensurable observations into a unified [0,1] Pareto competitive space.

#### 4.2.2. Performance Analysis of Single-Source Cross-Modal Mapping and Controllability-Quality Trade-Offs

In the process of evolving from single-modality mapping toward collaborative generation, this section aims to reveal the intrinsic trade-off relationship between generation quality and controllable precision across different technical paradigms. To systematically present the architectural capacities across diverse modalities, Table 3, Table 4 and Table 5 employ a standardized symbolic notation: ●, ◐, ○, and “−” denote full native support, partial accommodation, limited baseline support, and missing data, respectively.

To ensure rigorous academic transparency and prevent selective reporting bias, comprehensive evaluation metrics are exhaustively enumerated within these benchmarking tables (e.g., dual attributes represented as “Synergy: AIS/AIC”). However, for the mathematical formulation of the OMNP and the subsequent Pareto topological projections, dimensional reduction is strictly required to prevent multicollinearity. Therefore, we deliberately select the most universally reported metric in each evaluation cluster as the Primary Functional Proxy (e.g., utilizing AIS solely for $\Delta G_{Synergy}$ and FID solely for $\Delta G_{Quality}$ in the audio-driven domain). This methodological design guarantees mathematical consistency while projecting heterogeneous observations into an orthogonal 2D Pareto coordinate system. Utilizing this framework, the resulting Pareto scatter plots function directly as the standardized Controllability versus Quality curves requested for robust architectural benchmarking. It is crucial to define that ‘Controllability’ serves as the macroscopic umbrella term in this topology; it is mathematically proxied by Synergy for temporally interwoven audio tasks, and by Fidelity for structurally rigid 3D and neural-semantic BCI domains.

**Figure 24:**
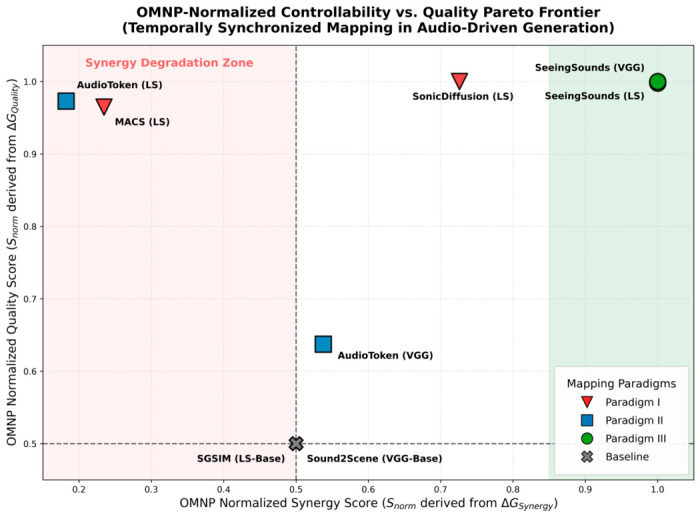
OMNP-Normalized Controllability vs. Quality Pareto Frontier for Audio-Driven Generation. The coordinate projection mathematically evaluates the trade-offs between temporal fidelity and generative quality, explicitly isolating the antagonistic degradations in Paradigm I architectures (e.g., MACS) and the holistic frontier expansion by Paradigm III (SeeingSounds). The red-shaded area indicates the Synergy Degradation Zone, whereas the green-shaded area represents the global Pa-reto optimal region.

**Audio-Driven Generation (Temporally Synchronized Mapping):** As illustrated in Table 3 and its corresponding OMNP-Normalized Pareto Frontier (Figure 24), audio-driven models are evaluated through their architectural capacities (Fidelity/Non-intrusive) alongside primary quantitative dimensions: Synergy (quantified via AIS and AIC) and Quality (quantified via FID and CLIP). The Pareto scatter plot categorizes the generative efficacy into distinct topological regions. Notably, Paradigm I architectures (e.g., MACS) and specific Paradigm II configurations (e.g., AudioToken on the Landscape dataset) fall strictly into the visually demarcated “Synergy Degradation Zone” ($S_{norm}<0.5$ on the normalized Synergy axis). This topological mapping explicitly reveals distinct structural capacity limits. For Paradigm I models like MACS, standard cross-attention bottlenecks struggle with the dense gradient competition induced by complex multi-source audio fusion. Parallelly, for Paradigm II models like AudioToken, forcing unstructured environmental audio (e.g., the Landscape dataset) into a frozen pseudo-text embedding space inherently triggers acoustic information loss. To maintain global visual quality under these respective structural constraints, both architectures are mathematically forced to compromise on absolute cross-modal alignment, resulting in antagonistic negative gains ($\Delta G_{Synergy}$ of −0.020 and −0.024, respectively). Conversely, Paradigm III models (e.g., SeeingSounds) effectively circumvent this trade-off. By achieving substantial positive relative gains in both perceptual Quality ($\Delta G_{Quality}$ up to +99.6) and audio-visual synchronization ($\Delta G_{Synergy}$ up to +0.235), SeeingSounds robustly occupies the upper-right global Pareto optimal region across heterogeneous datasets (VGGSound and Landscape). This mathematically validates its capacity to maximize Quality and Synergy simultaneously without inducing topological degradation.

**Figure 25:**
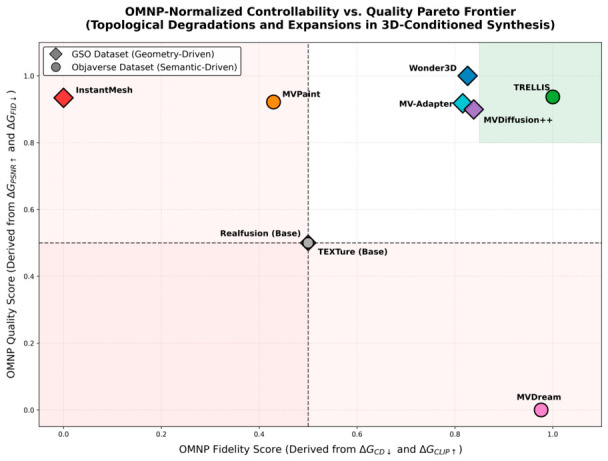
**OMNP-Normalized Controllability vs. Quality Pareto Frontier for 3D-Conditioned Synthesis.** Evaluated across geometry-driven (GSO) and semantic-driven (Objaverse) ecosystems, the topological mapping explicitly reveals the spatial-semantic gradient conflicts (evidenced by distinct degradation zones) and the robust arbitration by native 3D paradigms. The red-shaded area indicates the Fidelity Degradation Zone, while the green-shaded area represents the global Pareto optimal region. In the scatter plot, diamonds and circles denote the geometry-driven GSO and semantic-driven Objaverse datasets, respectively.

Three-dimensional Geometry-Conditioned Generation (Spatially Consistent Mapping): As detailed in Table 4 and its accompanying OMNP-Normalized Pareto scatter plot (Figure 25), architectures are evaluated across two disparate datasets—geometry-driven GSO and semantic-driven Objaverse. To ensure rigorous benchmarking while preventing multicollinearity during topological mapping, core capabilities are decomposed into two functional dimensions driven by dataset-specific Primary Proxies: Spatial Consistency (S.C.) and Semantic Generation (S.G.). Within the GSO ecosystem, S.C. evaluates physical rigidity utilizing Chamfer Distance (CD) for Fidelity and Peak Signal-to-Noise Ratio (PSNR) for Quality. Here, Wonder3D (Paradigm I) ensures high-fidelity S.C. through explicit constraints, achieving substantial positive relative gains in both geometric precision ($\Delta G_{Fidelity}$ of +0.0629) and signal quality ($\Delta G_{Quality}$ of +10.81). Conversely, InstantMesh suffers severe structural collapse, plunging into the “Fidelity Degradation Zone” with an antagonistic negative gain ($\Delta G_{Fidelity}$ of −0.0981). Parallelly, S.G. is evaluated within the Objaverse dataset, prioritizing conceptual alignment via CLIP for Fidelity and FID for Quality. Within this space, MVDream (Paradigm II) maximizes semantic priors ($\Delta G_{Fidelity}$ of +10.08) but crashes into the “Quality Degradation Zone” by sacrificing visual realism ($\Delta G_{Quality}$ of −11.01). To bridge this cross-domain divide and resolve these bipartite routing bottlenecks, the state-of-the-art TRELLIS (Paradigm IV) leverages structured 3D latent representations to support both S.C. and S.G. intrinsically. By securing robust positive gains across semantic alignment ($\Delta G_{Fidelity}$ of +11.50) and perceptual realism ($\Delta G_{Quality}$ of +7.78) without structural compromise, TRELLIS confidently anchors the global Pareto optimal frontier, marking a fundamental technical leap from early multi-view “image correction” to “native 3D generation.”

**Figure 26:**
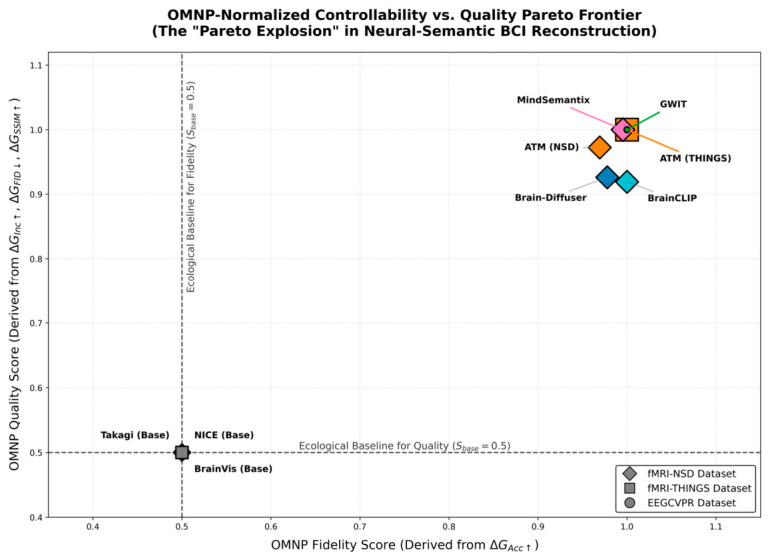
**OMNP-Normalized Controllability vs. Quality Pareto Frontier for Neural-Semantic BCI Reconstruction.** The unified mapping completely lacks degradation behaviors, formalizing the “Pareto Explosion” phenomenon where mapping discrete neural embeddings into the denoising manifold inherently circumvents dense topological interferences. In the scatter plot, diamonds, squares, and circles denote the fMRI-NSD, fMRI-THINGS, and EEG-CVPR datasets, re-spectively. The dashed lines represent the ecological baselines (S_base_ = 0.5) for Fidelity and Quality.

Neural-Semantic Reconstruction (BCI Signals): Table 5 synthesizes neural decoding architectures across diverse fMRI and EEG ecosystems, tracking cross-subject generalization (Gen.) and real-time processing capabilities (Real-time) as core architectural features. Within the corresponding OMNP-Normalized Pareto space (Figure 26), topological mapping relies on dataset-specific Primary Proxies to maintain dimensional integrity. Semantic Fidelity is universally governed by classification accuracy (Acc). Conversely, perceptual Quality ($\Delta G_{Quality}$) is mathematically derived from domain-aligned proxies, selected to reflect each dataset’s critical generative bottleneck:

(1) For static fMRI reconstructions (NSD/THINGS), the Inception metric (Inc, explicitly formulated as a two-way identification task evaluating the cosine similarity of Inception V3 feature vectors) is isolated, as it most rigorously quantifies the preservation of high-level semantic manifolds;
(2) For dynamic fMRI-Video domains, SSIM is selected to strictly enforce the spatiotemporal structural consistency required across continuous frames;
(3) For the highly noisy, low-spatial-resolution EEG signals, FID serves as the optimal proxy, directly penalizing distributional deviations to ensure global perceptual realism.

In stark contrast to the antagonistic gradient collisions endemic to 3D geometry-conditioned synthesis, the Brain–Computer Interface (BCI) domain exhibits a substantially different topological behavior, which we formalize as the “Pareto Explosion.” An analysis of the coordinate projections reveals a complete absence of models falling into any degradation zone. Instead, architectures leveraging advanced cross-attention and latent diffusion mechanisms register overwhelming positive trajectories. For instance, MindSemantix achieves massive synergistic expansions within the NSD ecosystem (yielding a $\Delta G_{Fidelity}$ of +17.3 alongside a $\Delta G_{Quality}$ of +17.8 derived from Inc), while GWIT radically pushes the optimal boundaries within the EEG domain (securing a $\Delta G_{Fidelity}$ of +42.0 and a $\Delta G_{Quality}$ of +40.55 derived from FID). Significantly, this mathematical framework strictly enforces empirical rigor; for example, within the fMRI-Video dataset, because the baseline (f-CVGAN) lacks a reported Accuracy metric, MinD-Video is explicitly excluded from the orthogonal 2D topological mapping to prevent speculative imputation, although it successfully registers a positive perceptual gain ($\Delta G_{Quality}$ of +0.058). This ubiquitous dual-dimensional growth mathematically proves that projecting sparse, discrete neural embeddings directly into the denoising manifold inherently bypasses the dense spatial interferences seen in other modalities, facilitating a holistic expansion of the global Pareto optimal frontier.

#### 4.2.3. Performance Analysis of Image Generation via Multi-Source Heterogeneous Collaboration and Conflict Regulation

While the single-modality mapping modules evaluated in Section 4.2.2 attain representational limits within their respective isolated domains, empirical observations indicate that generative performance is frequently bottlenecked by fixed gradient allocation strategies when confronting intertwined multi-source heterogeneous signals. Having validated the orthogonal Quality and Fidelity dimensions across single-modal conditions, we now explicitly activate the third macroscopic dimension of the OMNP framework—Stability (quantified by the Asymmetry Index, A)—to rigorously map these complex inter-conditional gradient interferences. Within this holistic 3D competitive topology, Vanilla ControlNet is established as the primary multi-source performance anchor (occupying the $S_{base}=0.5$ baseline across all axes).

**Figure 27:**
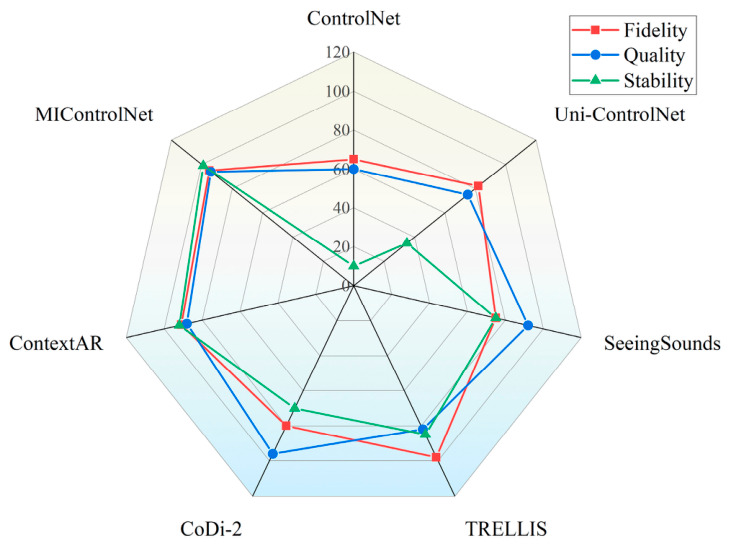
Performance game topology diagram under multi-source heterogeneous collaborative conflict regulation. The red, blue, and green lines represent the three macroscopic dimensions of the OMNP framework: Fidelity, Quality, and Stability, respectively.

As illustrated in Figure 27, the topological evolution of the performance envelope intuitively reflects generational technological leaps: early architectures, under strong conflict constraints, frequently experienced severe collapse along the Stability axis due to gradient field asymmetry (mathematically reflected as elevated A values), inducing a “tug-of-war effect.” In contrast, next-generation paradigms represented by MIControlNet [111] and ContextAR [155]—through precise arbitration of gradient interference (minimizing A)—achieve significant convex hull expansion, driving the Pareto frontier to migrate holistically toward the full-efficacy space and substantially enhancing the system’s non-dominance.

Building upon this systematic synthesis, this review distills scenario-specific deployment heuristics into a comprehensive Decision Matrix (Table 6). This mapping marks a formal transition of multi-source collaborative technology—from the empirical stage of naive modality stacking toward an engineering application stage centered on precision conflict arbitration. While Native Token Fusion models (e.g., FLUX.2) are temporarily absent from the quantitative envelope due to a lack of multi-source benchmark data, their topological advantages fundamentally circumvent bipartite routing bottlenecks, situating them as the ultimate theoretical paradigm for general-balanced scenarios.

## 5. Future Prospects

Although the preceding chapters have revealed significant advances in structural controllability and semantic alignment within cross-modal mapping and multi-source collaborative technologies, achieving a general-purpose controllable generation system characterized by strict physical consistency, deep semantic disentanglement, and real-time sensor-driven feedback remains a formidable challenge. Moving beyond pure algorithmic constraints, future generative frameworks must bridge the gap between high-dimensional latent spaces and the noisy, dynamic realities of physical sensor networks. Based on current technical boundaries, future research will be directed toward realizing a paradigm leap from “unidirectional static mapping” to “dynamic physical–digital collaboration,” with focused attention on the following six key directions:

(1) Geometric Dimension: Intrinsic Alignment via Riemannian Geometry. Existing paradigms largely rely on projecting heterogeneous signals into the prompt space of pre-trained models, resulting in pronounced topological distortion when handling raw, unaligned data from actual physical sensors (e.g., sparse LiDAR point clouds, continuous audio waveforms). The future challenge lies in leveraging Riemannian geometry or geometric deep learning (GDL) to achieve intrinsically consistent alignment between structured physical priors and the diffusion latent space, fundamentally eliminating spatial distortion [156]. Furthermore, there is an urgent need to construct a universal encoding foundation that enables the natural fusion of heterogeneous sensor signals at the underlying continuous feature level, thereby facilitating joint representational learning directly from raw physical inputs [157].
(2) Mechanistic Dimension: Structural Causal Models and Counterfactual Validation. To address the severe gradient competition and “semantic drift” arising from multi-source condition superposition, future resolution strategies must transition from static weighted fusion to explainable, causal mechanisms [158]. Specifically, future works should embed Structural Causal Models (SCMs) directly into the diffusion sampling trajectory. By applying *do*-calculus interventions during the reverse process to map the underlying relationships between distinct control signals [159], models can achieve true structural disentanglement. Experimentally, this paradigm must be validated through “Counterfactual Generation Protocols”—holding the initial noise seed constant while intervening on a single control variable—and utilizing metrics like localized LPIPS variance to strictly quantify the purity of feature decoupling, ensuring precise interventions without unintended structural collapse.
(3) Physical Dimension: PDE-Constrained Diffusion and Simulation Grounding. To overcome the pervasive “statistical simulation” characteristic of current controllable generation, future research must anchor generative outputs to real-world physics. This requires incorporating physical equations—such as Navier–Stokes equations for fluid dynamics or rigid body mechanics—as explicit regularization terms ($L_{PDE}$) within the diffusion process [160]. Through physics-informed generation, outputs will not only achieve visual realism but also strictly conform to environmental sensor data (e.g., gravity, collision, and depth layouts). Simultaneously, adapting these architectures for edge-device deployment is critical to meet the latency requirements of autonomous driving. Exploring acceleration techniques such as consistency models [161] and low-bit quantization [162] for real-time sensor-to-video inference remains a pressing open challenge.
(4) Frontier Domain: Efficient and Robust Mapping from Neural Manifolds to Visual Manifolds. As the ultimate frontier, Brain–Computer Interface (BCI) driven generation technology must overcome the challenges of real-world physiological sensors: extremely low signal-to-noise ratios and motion artifacts. Future research should prioritize cross-individual zero-shot generalization techniques with robust noise-filtering capabilities [163]. Leveraging large-scale neural representation pre-training will be essential for high-fidelity intent decoding without user-specific calibration. Moreover, constructing a real-time closed-loop interactive system—where physiological signals serve as instantaneous evaluation factors to correct the model’s personalized style—will be the key to realizing truly immersive human–machine symbiosis.
(5) Systemic Dimension: Hardware-in-the-Loop (HIL) and Embodied Metrics. As generative paradigms evolve toward multi-sensor integration, evaluation frameworks must advance from purely visual metrics to comprehensive, system-level protocols. Building upon the benchmarks proposed in Section 4.1, future work should establish Pareto-optimal evaluation standards [126] that measure multimodal alignment precision, quality, and latency. Crucially, Hardware-in-the-Loop (HIL) testing must be introduced by deploying optimized DiT architectures onto edge-computing devices (e.g., NVIDIA Jetson Orin) connected to simulators [164]. Instead of visual fidelity, experimental validation must prioritize “Task Success Rate” (TSR) and structural robustness under sensor noise to assess the true credibility of 4D scenarios [165]. On the ethical dimension, provenance analysis and robust safety filtering mechanisms for non-visual signal-driven content must be institutionalized [166] to prevent neural intent leakage and misuse.
(6) Cognitive Dimension: Fully Autonomous Agents and Lifelong Self-Correction. Building upon the MM-Agent routing mechanisms discussed in Section 3, the final trajectory of controllable generation is the evolution from static layout routing to dynamic autonomous ecosystems [167]. Future frameworks will integrate large reasoning models (System 2) with high-fidelity rendering backbones (System 1) into a continuous learning loop. In this paradigm, Vision-Language Models (VLMs) will act as persistent ‘Evaluator Agents’ that autonomously monitor outputs against physical compliance rules. When deviations occur, the agentic system will autonomously rewrite control codes and adjust conditioning parameters for iterative self-correction, representing the zenith of general-purpose controllable generation.

## Figures and Tables

**Table 1:** Comprehensive taxonomy and quantitative overhead comparison of representative cross-modal mapping methods.

Method	Input Modality	Mapping Strategy	Injection Mechanism	Backbone	Control Granularity	Overhead (Params/Δθ/VRAM)	Application Scenario
SonicDiffusion [42]	Audio	Structured Token	Parallel Cross-Attn	U-Net	Acoustic Rhythm	~50 M/~+5.8%/~8 GB	Audio-to-Image
AudioToken [58]	Audio	Pseudo-text Token	Text Cross-Attn	U-Net	Semantic Attribute	<1 M/~0.0%/~6 GB	Sound-guided Gen
SeeingSounds [84]	Audio	Dual-Space	Bypass Modulation	U-Net/DiT	Temporal Sync	~45 M/~+5.2%/~8 GB	Dynamic Acoustics
Wonder3D [89]	3D (Maps)	Explicit Geometry	Zero-Conv Residual	U-Net	Geometric Topology	~20 M/~+2.3%/~10 GB	Multi-view Synth
MVDream [94]	3D (Pose)	Parametric Encode	Decoupled Cross-Attn	U-Net	Viewpoint & Semantic	0 M (Full Tune)/0.0%/~12 GB	Text-to-3D Gen
InstantMesh [97]	3D (Image)	Multi-View Extractor	Epipolar Bias Attn	U-Net/DiT	Spatial Ray	~150 M/~+17.4%/~16 GB	3D Mesh Recon
TRELLIS [102]	3D (Mesh)	Structured Latent	3D Denoising	DiT	Physical Coherence	Native DiT/N/A/~16 GB	3D Asset Gen
MindSemantix [106]	fMRI (BCI)	Brain-to-Text	Text Cross-Attn	U-Net	Abstract Concept	~15 M/~+1.7%/~6 GB	Concept Recon
DreamDiffusion [108]	EEG (BCI)	Latent Regression	Semantic Injection	U-Net	Category Intent	~20 M/~+2.3%/~6 GB	EEG-to-Image
MIControlNet [111]	Multi-src	Modality Adapters	Orthogonalization	U-Net	Complex Spatial	~722 M (2×)/+84.0%/>12 GB	Precision Scene
FreeControl [112]	Multi-src	Score Guidance	Gradient Projection	U-Net/DiT	Spatial-Semantic	0 M (Train-Free)/0.0%/~14 GB	Zero-shot Control
OminiControl [73]	Multi-src	Unified Token	Joint Self-Attn	DiT	Global & Local	14.5 M/+0.12%/~24 GB	Universal Image

**Table 2:** Systemic Comparison of U-Net and DiT Architectures in Multi-Source Collaboration.

Comparison Dimension	U-Net Pathway	DiT Pathway
Representational Base	Continuous 2D spatial feature maps	Discrete 1D patchified token sequences
Cross-Modal Injection	Asymmetric Attention & Bypass Addition	Sequence Concatenation & Self-Attention
Computational Complexity	$O(M\cdot N_{img}\cdot N_{cond}\cdot d)$ (Linear scaling with modality count M)	$O((N_{img}+\sum_{i=1}^{M}N_{cond,i}{)}^{2}\cdot d)$ (Quadratic with total sequence length)
Scalability Paradigm	Structural Expansion (Requires parallel encoders per condition)	Unified Manifold (Native fusion, minimal overhead)
Primary Bottleneck	Capacity Saturation & VRAM Exhaustion (Semantic overwrite across layers)	Attention Weight Competition (Condition neglect via token imbalance)

**Table 3:** Quantitative Benchmarking of Audio-driven Models for Temporally Synchronized Mapping and Injection.

Models	Core Datasets	Arch. Features (Fidelity/Non-Intrusive)	Synergy (AIS ↑/AIC ↑)	ΔG_Synergy_	Quality (FID ↓/CLIP ↑)	ΔG_Quality_
SGSIM [143]	Landscape [85]	Baseline	0.722/0.217	0	220.3/-	0
AudioToken [58]	Landscape	○/●	0.698/0.285	−0.024	141.3/-	+79.0
MACS [80]	Landscape	○/○	0.702/0.471	−0.02	147.2/-	+73.1
SonicDiffusion [42]	Landscape	●/○	0.739/0.544	+0.017	118.6/-	+101.7
SeeingSounds [84]	Landscape	●/●	0.760/0.791	+0.038	120.7/-	+99.6
Sound2Scene [144]	VGGSound [136]	Baseline	0.436/0.407	0	17.97/-	0
AudioToken	VGGSound	○/●	0.452/0.413	+0.016	17.1/0.78	+0.87
SeeingSounds	VGGSound	●/●	0.671/0.916	+0.235	9.2/0.91	+8.77

Note: ●, ○, and - denote full native support, partial accommodation, limited baseline support, and missing data, respectively. The symbols (↑) and (↓) indicate whether a higher or lower metric value signifies better generative performance.

**Table 4:** Quantitative Benchmarking of 3D Geometry-conditioned Models for Spatially Consistent Generation.

Models	Core Datasets	Arch. Features (S.C./S.G.)	Fidelity (CD ↓/CLIP ↑)	ΔG_Fidelity_	Quality (PSNR ↑/FID ↓)	ΔG_Quality_
Realfusion [145]	GSO [139]	Baseline	0.0819/-	0.00	15.26/-	0.00
Wonder3D [89]	GSO	●/○	0.019/-	+0.0629	26.07/-	+10.81
MV-Adapter [95]	GSO	◐/●	0.021/-	+0.0609	22.13/-	+6.87
InstantMesh [97]	GSO	○/●	0.180/-	−0.0981	22.79/-	+7.53
MVDiffusion++ [146]	GSO	●/◐	0.0165/-	+0.0654	21.45/-	+6.19
TEXTure [147]	Objaverse [138]	Baseline	-/20.30	0.00	-/28.03	0.00
MVPaint [90]	Objaverse	●/◐	-/19.87	−0.43	-/20.89	+7.14
MVDream [94]	Objaverse	◐/●	-/30.38	+10.08	-/39.04	−11.01
TRELLIS [102]	Objaverse	●/●	-/31.80	+11.50	-/20.25	+7.78

Note: ●, ◐, ○, and - denote full native support, partial accommodation, limited baseline support, and missing data, respectively. The symbols (↑) and (↓) indicate whether a higher or lower metric value signifies better generative performance.

**Table 5:** Quantitative Benchmarking of Neural-semantic Reconstruction Models based on BCI Signals.

Models	Core Datasets	Arch. Features (Gen./Real-Time)	Fidelity (Acc ↑)	ΔG_Fidelity_	Quality (fMRI: SSIM ↑/PixCorr ↑/Inc ↑) (EEG: FID ↓/IS ↑)	ΔG_Quality_
Takagi et al. [61]	fMRI-NSD [140]	Baseline	77.00%	0	-/-/76.0	0
Brain-Diffuser [104]	fMRI-NSD	○/○	92.50%	+15.5	0.367/0.305/87.2	+11.2
MindSemantix [106]	fMRI-NSD	◐/●	94.30%	+17.3	0.333/0.299/93.8	+17.8
BrainCLIP [109]	fMRI-NSD	◐/●	94.80%	+17.8	-/-/86.7	+10.7
ATM [148]	fMRI-NSD	●/●	91.70%	+14.7	0.366/0.305/91.0	+15.0
NICE [149]	fMRI-THINGS [150]	Baseline	72.20%	0	0.276/0.142/65.9	0
ATM	fMRI-THINGS	●/●	78.60%	+6.4	0.345/0.160/73.4	+7.5
f-CVGAN [151]	fMRI-Video [152]	Baseline	-	-	0.118/-/-	0
MinD-Video [105]	fMRI-Video	○/○	85.30%	-	0.176/-/-	+0.058
BrainVis [153]	EEG-CVPR [154]	Baseline	49.00%	0	121.02/31.52	0
GWIT [110]	EEG-CVPR	●/●	91.00%	+42.0	80.47/33.32	+40.55

Note: ●, ◐, ○, and - denote full native support, limited baseline support, and missing data, respectively. The symbols (↑) and (↓) indicate whether a higher or lower metric value signifies better generative performance.

**Table 6:** Technology Selection Decision Matrix for Multi-Source Collaborative Generation Based on Performance Game Characteristics.

Application Scenario	Core Performance Priorities	Representative Game-Theoretic Characteristics	Recommended Paradigms & Representative Models
Precision-Oriented	Geometric Fidelity and Physical Consistency	Expansion along Fidelity and Stability axes; high geometric rigidity.	Explicit Gradient Arbitration: MIControlNet [111], TRELLIS [102]
Semantic-Creative	Perceptual Quality and Semantic Expressiveness	Maximal shift toward the Quality axis; high flexibility in structural constraints.	Implicit Semantic Mapping: CoDi-2 [142], SeeingSounds [84]
General-Balanced	Full-Modality Compatibility & Robustness	Full-dimensional expansion of the Pareto front; balanced and near-ideal envelope.	Autoregressive Robust Architecture: ContextAR [155], MIControlNet, FLUX.2 [118], OminiControl [73]

## Data Availability

The original contributions presented in the study are included in the article; further inquiries can be directed to the corresponding author.

## Abbreviations

The following abbreviations are used in this manuscript:

Abbreviation	Full Name
BCI	Brain–Computer Interface
DiT	Diffusion Transformer
EEG	Electroencephalography
fMRI	Functional Magnetic Resonance Imaging
HIL	Hardware-in-the-Loop
k-NN	k-Nearest Neighbors
LDM	Latent Diffusion Model
LLM	Large Language Model
MLP	Multi-Layer Perceptron
NeRF	Neural Radiance Field
PDE	Partial Differential Equation fMRI
SNR	Signal-to-Noise Ratio
T2I	Text-to-Image
VAE	Variational Autoencoder
VDVAE	Very Deep Variational Autoencoder
